# The down-regulation of XBP1, an unfolded protein response effector, promotes acute kidney injury to chronic kidney disease transition

**DOI:** 10.1186/s12929-022-00828-9

**Published:** 2022-06-28

**Authors:** Jia-Huang Chen, Chia-Hsien Wu, Jia-Rong Jheng, Chia-Ter Chao, Jenq-Wen Huang, Kuan-Yu Hung, Shing-Hwa Liu, Chih-Kang Chiang

**Affiliations:** 1 Graduate Institute of Toxicology, College of Medicine, National Taiwan University, No.1 Jen Ai road section 1, Taipei, 100 Taiwan; 2 Department of Internal Medicine, College of Medicine, National Taiwan University, Taipei, Taiwan; 3 Department of Integrated Diagnostics and Therapeutics, National Taiwan University Hospital, Taipei, Taiwan; 4 Center for Biotechnology, National Taiwan University, Taipei, Taiwan

**Keywords:** Unfolded protein response, XBP1, Fibrosis, Acute kidney injury, Chronic kidney disease

## Abstract

**Background:**

The activation of the unfolded protein response (UPR) is closely linked to the pathogenesis of renal injuries. However, the role of XBP1, a crucial regulator of adaptive UPR, remains unclear during the transition from acute kidney injury (AKI) to chronic kidney disease (CKD).

**Methods:**

We characterized XBP1 expressions in different mouse models of kidney injuries, including unilateral ischemia–reperfusion injury (UIRI), unilateral ureteral obstruction, and adenine-induced CKD, followed by generating proximal tubular XBP1 conditional knockout (XBP1^cKO^) mice for examining the influences of XBP1. Human proximal tubular epithelial cells (HK-2) were silenced of XBP1 to conduct proteomic analysis and investigate the underlying mechanism.

**Results:**

We showed a tripartite activation of UPR in injured kidneys. XBP1 expressions were attenuated after AKI and inversely correlated with the severity of post-AKI renal fibrosis. XBP1^cKO^ mice exhibited more severe renal fibrosis in the UIRI model than wide-type littermates. Silencing XBP1 induced HK-2 cell cycle arrest in G2M phase, inhibited cell proliferation, and promoted TGF-β1 secretion. Proteomic analysis identified TNF receptor associated protein 1 (Trap1) as the potential downstream target transcriptionally regulated by XBP1s. Trap1 overexpression can alleviate silencing XBP1 induced profibrotic factor expressions and cell cycle arrest.

**Conclusion:**

The loss of XBP1 in kidney injury was profibrotic, and the process was mediated by autocrine and paracrine regulations in combination. The present study identified the XBP1-Trap1 axis as an instrumental mechanism responsible for post-AKI fibrosis, which is a novel regulatory pathway.

**Supplementary Information:**

The online version contains supplementary material available at 10.1186/s12929-022-00828-9.

## Background

Acute kidney injury (AKI) is featured by an abrupt loss of kidney function, associated with high morbidity and mortality, especially in hospitalized patients [1]. Evidence suggests that AKI contributes to an increased risk of long-term cardiovascular events, fractures, infections as well as the progression of chronic kidney disease (CKD) and end-stage renal disease (ESRD) development [2–5]. Clinically, ischemia–reperfusion injury (IRI) is the most common etiology of AKI, involving renal tubular injury and death [6, 7]. Aberrant tubular repair may be a critical promoter for the transition from AKI to CKD [4, 8, 9]. Maladaptive repaired proximal tubular cells adopt a partial epithelial-mesenchymal transition (partial EMT) phenotype, which is characterized by cell cycle arrest in the G2/M phase, the activation of transcription factor Snai1, and secretion of profibrotic factors: transforming growth factor β1 (TGFβ1) and connective tissue growth factor (CTGF) [9–11]. Intriguingly, TGFβ1 stimulates G2/M arrest and the production of profibrotic cytokines in tubular cells, leading to perivascular fibroblasts pericyte-myofibroblast trans-differentiate into myofibroblasts and contributing to renal fibrosis in various animal models of AKI-induced fibrosis [12, 13]. Mechanisms responsible for AKI to CKD transition are complex and involve apoptosis, autophagy, cell cycle arrest, inflammation, ER stress, mitochondria dysfunction, and senescence. Despite this complexity, there is still an unmet medical need in uncovering potential therapeutic targets for patients with AKI to CKD transition.

Endoplasmic reticulum (ER) is responsible for protein synthesis, processing, and transport, and it is essential for maintaining cellular homeostasis. Insults disturbing ER functions lead to ER stress, followed by the induction of unfolded protein response (UPR) which is crucial for maintaining ER proteostasis [14]. Dysregulated ER is associated with disease progression, especially for renal fibrosis, which has been reported in many studies ER stress is involved in AKI to CKD transition [15–18].

During ER stress, three transmembrane stress sensors PKR-like ER kinase (PERK), activating transcription factor 6 (ATF6) and inositol-requiring enzyme 1α (IRE1α) are activated to regulate protein synthesis, degradation, and folding capacity. Activation of the PERK-eIF2α axis inhibits global protein translation while paradoxically inducing the selective translation of ATF4 and the activation of the integrated stress response [19]*.* ATF6 is proteolytically activated by site-1 and site-2 proteases (S1P and S2P) in Golgi apparatus [20], and the released transcriptionally active N-terminal domain upregulates genes encoding ER chaperones involved in ER-associated protein degradation (ERAD) [21]. IRE1α activation undergoes conformational changes to promote its dimerization, oligomerization, trans-autophosphorylation, RNase activation and eventually leads to unconventional splicing 26 nucleotides of X-box binding protein 1 (XBP1) mRNA to produce spliced XBP1 (XBP1s). XBP1 mRNA ordinarily translates into an unspliced isoform of XBP1 (XBP1u) and is spliced into the transcription factor XBP1s during ER stress stimulation. Activated XBP1s function as an effector protein response for regulating UPRs targeting gene by elevating protein folding capacity, ER chaperones expression, ER expansion and ERAD regulation [22, 23]. XBP1s also determines cell fate via relieving ER stress or initiating apoptosis in the presence of irreversible ER stress, whereas XBP1u, encoded from unspliced XBP1 pre-mRNA, regulates the stability, expression and transcriptional activity of XBP1s [24–26].

Although the activation of UPR has been implicated in the pathogenesis of diabetic nephropathy, congenital nephrotic syndrome, and interstitial renal fibrosis [16, 17, 27], the connection between UPR and profibrogenic signaling has not been well explored. Our previous work demonstrated that overwhelming ER stress was significantly activated in the unilateral ureteral obstruction (UUO)-induced renal fibrosis and coincident with attenuation of XBP1 expression, adaptive UPR effector [17]. In order to explore the potential therapeutic role of adaptive UPR in renal fibrosis, we further study the critical molecule XBP1 in the AKI to CKD transition. The decreased tubular XBP1 expression enhances post-AKI fibrosis by inducing DNA damage response and consequent cell cycle G2/M arrest, followed by the activation of profibrotic signaling.

## Materials and methods

### Mice

*XBP1*^*fl/fl*^ mice were kindly provided by Dr. Laurie H. Glimcher (Weill Cornell Medicine, New York, NY, USA) and *Slc5a-CreERT2* mice were purchased from National Laboratory Animal Center-Rodent Model Resource Center (Taipei, Taiwan). XBP1 proximal tubular conditional knockout mice are generated by breeding the *Slc5a-CreERT2* mice with a *XBP1*^*fl/fl*^ mice. Genotyping was performed on genomic DNA extracted from toes by PCR with specific primers listed in Additional file 1: Table S1. All animal experiments were approved by National Taiwan University College of Medicine and College of Public Health Institutional Animal Care and Use Committee (IACUC No. 20150030, 20170540). Experimental protocols and animal care were provided according to the guideline for the care and use of animals established by National Taiwan University.

### Animal models

UUO, unilateral ischemia–reperfusion injury (UIRI), and adenine-induced nephropathy were used in this study. UUO, bilateral ischemia–reperfusion injury (BIRI), and UIRI with contralateral nephrectomy (UIRI + Nx) are commonly used animal models for studies of ischemic AKI and post-AKI fibrosis. Recently, it is reported that UIRI without contralateral nephrectomy (hereafter described as UIRI) induces renal pathology closely resembling those of patients with nephropathy [28]. With optimized control of animal’s body temperature during ischemia, UIRI causes long-term expression of tubular injury markers, fibrosis-related genes, and the deposition of extracellular matrix, suggesting that UIRI can be useful for studying post-AKI fibrosis. C57BL/6 mice aged from 10 to 12 weeks were anesthetized and subjected to 30 min left kidney UIRI. The left kidney of the euthanized animal was clamped for 30 min at 37.5 °C, followed by sera and tissue collection at 1, 3, 5, 11 and 15 days after UIRI (Additional file 2: Fig. S1a). Mice body temperature were monitored by a homeothermic monitor system (Physitemp Instruments Inc., Clifton, NJ, USA) and maintained at 37.5 °C during surgery. For UUO model, the left ureter was obstructed by two-point ligations at the ureteropelvic junction with 4–0 silk sutures through a left flank incision. The sham group underwent the same surgical procedure without clamping renal pedicles or ureter ligation. All animals were sacrificed after 7 days of UUO. For adenine induced CKD, the regular chew diet of LabDiet 5001 (TestDiet) containing 0.25% adenine was purchased from Bio-cando incorporation (Taoyuan, Taiwan). Chronic adenine nephropathy was induced by feeding animals with or without 0.25% adenine diet alternately for 5 weeks (2 weeks adenine diet then 1 week chow diet for rest). Mice receiving regular chow served as the control group. For UIRI with contralateral nephrectomy (UNx) model performed in XBP1 transgenic mice, all mice subjected to 30 min of UIRI then conduct contralateral nephrectomy before 1 day of sacrifice and sacrificed at day 15. Mice serum were collected to detect serum creatinine (Scr) and blood urea nitrogen (BUN) levels using FUJI DRI-CHEM 4000i (Fujifilm, Tokyo, Japan).

### Reagents and antibodies

The following antibodies were used: anti-phospho-IRE1α (NB100-2323) was from Novus Biologicals (Littleton, CO, USA); anti-IRE1α (#3294) and anti-γH2AX (#2577) were from Cell Signaling Technology (Danvers, MA, USA); anti-phospho-PERK (SC-32577), anti-PERK (SC-13073), anti-p21 (SC-6246), anti-Chk1 (SC-8408), anti-Cyclin B1 (SC-245), anti-Cyclin D1 (SC-246) and anti-XBP1 (SC-7160) for detecting XBP1s in animal model were from Santa Cruz Biotechnology (Dallas, TX, USA); anti-H2AX (ab124781) and anti-XBP1 (ab37152) for detecting XBP1u in the animal model and in HK-2 cells were from Abcam (Cambridge, UK); anti-α-smooth muscle actin (GTX100034), anti-Trap1 (GTX102017) and anti-vimentim (GTX100619) were from Genetex Inc. (Irvine, CA, USA); anti-GAPDH (G8795) was from Sigma-Aldrich (St. Louis, MO, USA); anti-ATF6 was kindly provided by Dr. Jim-Tong Horng (Chang Gung University, Taiwan) [29]. Anti-mouse IgG (H + L) and anti-rabbit IgG (H + L) antibodies were purchased from Genetex. The XBP1 siRNA (5′-CCUUGUAGUUGAGAACCAGGAGUUA-3′) and siNTC (medium GC of Stealth negative control duplex) were purchased from Thermo Fisher Scientific (Waltham, MA, USA) and transfected into cells with TransIT-X2 (Mirus Bio, Madison, WI, USA) according to the manufacturer’s manual. The Smart Quant Green master mix for the real-time quantitative assay was purchased from Protech (Taipei, Taiwan). Human TGFβ1 Detection enzyme-linked immunosorbent assay (ELISA) kit was purchased from R&D Systems (Minneapolis, MN, USA) and used according to the manufacturer’s instructions.

### Cell culture

HK-2 and 293 T cells were obtained from Bioresource Collection and Research Center (Hsinchu City, Taiwan) and were cultured in Dulbecco’s Modified Eagle Medium (DMEM): F12 (1:1) or DMEM medium (Thermo Fisher Scientific) supplemented with 10% fetal bovine serum (FBS; Thermo Fisher Scientific) at 37 °C in a 5% CO_2_-humidified environment.

### Plasmid construction and cell transfection

The XBP1s and Trap1 were amplified by PCR from the cDNA prepared from HK-2 cells using the following primers. For XBP1s: 5′-ATCggCTAgCATggATTATAAggATgACgACgATAAAgTggTggTggCAgCCgCg-3′, 5′-CgATgCTAgCTTAgACACTAATCAgCTggggAAAgAgTTC-3′. The product was inserted into the Nhel sites of pLKO-AS3w-puro (Academia Sinica, Taipei, Taiwan). For Trap1: 5′-ATTAggTACCATggCgCgCgAgCTgCg-3′, 5′-ATgAgAATTCCAgTgTCgCTCCAgggCC-3′. The product was inserted into the KpnI and EcoR1 sites of pcDNA3.1(+)/myc-His B (Thermo Fisher Scientific). For the transfection conditions, 40 nM of siRNA and 1 μg of plasmid for a 35 mm dish was premixed with TransIT-X2 reagent (Mirus Bio) according to the manufacturer’s protocol.

### Conditioned medium collection and procedure

The conditioned medium was collected from HK-2 cells silenced of XBP1 for 48 h. After collecting the supernatants, the conditional medium was transferred to culture 293 T cells for 48 h.

### Histological analysis

For histology assessment, fixed kidneys were paraffin-embedded and sectioned into 4-μm sections and were stained with Periodic Acid-Schiff (PAS) and Masson’s trichrome to estimate renal histological injury and renal fibrosis. For PAS staining, deparaffinized and rehydrated renal sections were stained with 0.5% periodic acid for 5 min and then stained with Schiff reagent for 15 min, followed by counterstaining with hematoxylin solution for 1 min. For Masson’s trichrome staining, deparaffinized and rehydrated sections were stained with Bouin’s fixative overnight and then stained with Weigert’s iron hematoxylin solution and Biebrich scarlet-acid fuchsin solution. After incubating in phosphomolybdic–phosphotungstic acid solution, collagen was determined by staining with aniline blue. The collagen-stained renal sections were quantified by ImageJ software (NIH, http://rsbweb.nih.gov/ij/). In brief, the red channel of RGB stack images was selected to set the blue-stained fibrosis area and the whole tissue area separately. The blue-stained area to cross the whole image is represented as fibrosis fraction for the degree of interstitial collagen deposition. Fifteen cortical tubulointerstitial fields that were randomly selected at 200× magnification were assessed in each mouse, and the average for each group was analyzed. The images were acquired using an Olympus BX51 microscope equipped with an Olympus DP72 camera and cellSens Standard 1.14 software (Olympus, Tokyo, Japan).

### TUNEL assay

The DeadEndTM Fluorometric TUNEL System (Promega, Madison, WI, USA) was used for detecting DNA damage in renal tissue. The procedure followed the manual description. Briefly, the paraffin-embedded tissue slide was deparaffinized and rehydrated. After fixing with 4% paraformaldehyde for 15 min, the tissue section was incubated with proteinase K and then incubated with the nucleotide mixture containing fluorescein-12-dUTP and terminal deoxynucleotidyl transferase. 200 × magnification fields were randomly captured, and the counts of fluorescent DNA damage cells were counted.

### Gene expression analysis

Total RNAs of fresh kidney samples were extracted with TRIzol reagent (Life Technologies, Grand Island, NY, USA) according to the manufacturer’s protocol. Total RNA of HK2 cells were extracted using GENEzol™ TriRNA Pure Kit (Geneaid, New Taipei City, Taiwan). After DNase treatment, 1 μg of RNA was reverse transcribed with iScript reverse transcription supermix (Bio-Rad, Hercules, CA, USA). The resulting cDNA products were amplified with specific primer pairs to detect mRNA abundance using a StepOnePlus real-time polymerase chain reaction (PCR) system (Thermo Fisher Scientific). The expressions of the target genes were calculated using the relative comparative quantitation method. The sequences of the primer pairs are listed in Additional file 1: Table S1.

### Western blotting

Cells were lysed with RIPA lysis buffer (Cell Signaling Technology), and cell debris were removed by centrifugation at 14,000 rpm for 10 min at 4 °C. The protein concentrations were measured by the Coomassie Protein Assay Reagent (Thermo Fisher Scientific). Equal amounts of proteins (15–30 μg) were subjected to sodium dodecyl sulfate polyacrylamide gel electrophoresis (SDS-PAGE) and transferred to the polyvinylidene difluoride membrane (Millipore, Billerica, MA, USA). The membrane was blocked with 5% non-fat milk in Tris-buffered saline-Tween (TBST) (0.2% Tween 20 (vol/vol) for 1 h followed by incubating with specific primary antibodies overnight. After washing with PBST, the membrane was incubated with horseradish peroxidase (HRP)-conjugated secondary antibodies and developed with Immobilon Western HRP Chemiluminescent Substrate (Millipore). The chemiluminescent image was captured with the BioSpectrum 810 Imaging System (UVP, Upland, CA, USA).

### Immunofluorescence staining

Fresh renal tissue was dehydrated with 30% sucrose for 24 h at 4 °C, then embedded with OCT gel. The 4-μm-thick tissue sections were further permeabilizated and blocked with the mixture of 0.1% saponin (Sigma-Aldrich), 1% gelatin (Sigma-Aldrich), 1% BSA (Sigma-Aldrich) or Mouse on Mouse Blocking Reagent (MKB-2213–1, Vector Laboratories, Burlingame, CA, USA) in the PBS for 1 h at room temperature. The primary antibody XBP1 (ab37152, Abcam), phospho-Histone H3 (ab14955, Abcam), and Ki67 (ab15580, Abcam) were incubated at 4 °C overnight. After three times of wash, the renal sections were incubated with Alexa Fluor 568-labeled anti-rabbit (# A-11011, Invitrogen, Carlsbad, CA) or Alexa Fluor 488-labeled anti-mouse (#4408, Cell Signaling Technology) secondary antibodies at room temperature for 1 h. Nuclei were stained with DAPI. The sections were then washed and mounted with ProLong Gold (#9071, Cell Signaling Technology). The images were captured using Leica Dmi8 fluorescence microscopy (Wetzlar, Germany).

### MTS [3-(4,5-dimethylthiazol-2-yl)-5-(3-carboxymethoxyphenyl)-2-(4-sulfophenyl)-2H-tetrazolium] assay

The cell growth and proliferation ability were measured with CellTiter 96^®^ Aqueous One Solution Cell Proliferation kit (Promega). The detailed procedure was described as previous [30]. Briefly, HK-2 cells were plated in a 96-well plate. After washing cells with PBS, a fresh culture medium containing 0.2% MTS was added to each well and incubated at 37 °C for 45 min. The absorbance was measured at 490 nm and 650 nm on the SpectraMax^®^ ABS Plus Microplate plate reader (Molecular devices, San Joes, CA, USA). Data were normalized to controls and represented as the proliferation rate of the controls.

### Flow cytometric analysis of cell cycle

After washing twice with ice-cold PBS, cells were collected and fixed using 70% ethanol at 4 °C for at least 30 min. Then the cells were washed twice with ice-cold PBS and stained with 50 µg/ml propidium iodide (P4170, Sigma-Aldrich) in the presence of 30 µg RNase A (Thermo Fisher Scientific) at room temperature for 30 min prior to analysis using flow cytometry (BD LSRII, BD bioscience, San Jose, CA, USA) with excitation laser 561 nm and bandpass filter 670/30. Data was output as FACS files and analyzed by FlowJo software (BD bioscience).

### Protein identification by LC–MS/MS analysis

Proteomic analysis technical was performed by BIOTOOLS Co., Ltd (New Taipei City, Taiwan). For sample preparation, in-sol digestion was applied. Each sample were first diluted in 100 mM triethylammonium bicarbonate (TEABC), and then reduced with 5 mM tris-(2- carboxyethyl)-phosphine (Sigma-Aldrich), followed by cysteine-blocking with 55 mM iodoacetamide (Sigma-Aldrich). After samples were digested with sequencing-grade modified porcine trypsin (Promega), the peptides were then labeled with dimethyl reagent, pooled, and desalted by homemade C18-microcolumn. Digested peptides were detected through LC–MS/MS analysis (Agilent Technologies, Santa Clara, CA, USA) with reverse column (Zorbax 300SB-C18, 0.3 × 5 mm, Agilent Technologies). The LC apparatus coupled with a 2D linear ion trap mass spectrometer (Orbitrap Classic, Thermo Fisher Scientific) and operated using Xcalibur 2.0.7 software (Thermo Fisher Scientific). For protein identification, the SwissProt database (released on Mar 16, 2016, extracted for Homo sapiens, 20,199 sequences) was searched using the Mascot search engine (Matrix Science, London, UK; version 2.5).

### Luciferase reporter assay

Each well of 24-well plate contained 4 × 10^4^ cells transfected with 0.4 μg of Trap1 promoter containing pGL3 luciferase reporter vectors (pGL3-basic) and 0.04 μg of thymidine kinase promoter-Renilla luciferase reporter plasmid (pRL-TK) as an internal control. Trap1 promoter region (− 1315 ~ + 54) is amplified by PCR using primers with built-in restriction sites (Additional file 1: Table S1). The PCR products are digested with NheI and HindIII, and ligated to a NheI and HindIII digested pGL3-basic plasmid. Cells are lysed and assayed for firefly and Renilla luciferase activity using the dual-luciferase kit (BIOTOOLS). The results are normalized to the Renilla luciferase activity of the internal control.

### Chromatin immunoprecipitation (ChIP) assay

Chromatin immunoprecipitation assay was performed using SimpleChIP^®^ Enzymatic Chromatin IP Kit (#9003, Cell Signaling Technology). Briefly, approximately 4 × 10^6^ cells were fixed with 1% formaldehyde for 10 min at room temperature then digested with Micrococcal Nuclease and sonicated after 2 sets of 6-s pulses using a VirTis Virsonic 100 Ultrasonic Sonicator at 50% amplitude with a 1/8-inch probe. The DNA–protein complex was immunoprecipitated using anti-XBP1 or anti-IgG antibody overnight at 4 °C. After eluting and purifying, XBP1s binding sequence was evaluated by PCR amplification using Trap1 primer sets as listed in Additional file 1: Table S1.

### Statistics

Results are presented as the mean ± standard error of the mean (SEM). Statistical significance of differences was determined by the Student’s two-tailed *t*-test or one-way ANOVA with Duncan post-test. A P-value of < 0.05 was interpreted as significant. All experiments were repeated at least three times to ensure reproducibility.

## Results

### Renal unilateral ischemia–reperfusion injury (UIRI) leads to post-injury fibrosis in mice

First, we confirmed the pathological findings of post-AKI injury in our UIRI model. Periodic acid–Schiff (PAS) staining found the accumulation of debris in the tubular lumen beginning at day 1 post-UIRI (Additional file 2: Fig. S1b). In addition, the expressions of AKI marker, kidney injury molecule-1 (Kim-1), increased with a peak at day 1 post-UIRI and gradually decreased over time (Additional file 2: Fig. S1c). The fibrotic fraction visualized by Masson’s trichrome staining showed a 30% increase compared to sham group at day 15 post-UIRI (Additional file 2: Fig. S1d-e). Consistently, the expression of α-SMA increased prominently throughout the course of the experiments (Additional file 2: Fig. S1f-g). In summary, these results demonstrate that UIRI causes prominent renal damage and the development of fibrosis.

### Activation and modulation of UPR in UIRI model

**Fig. 1:**
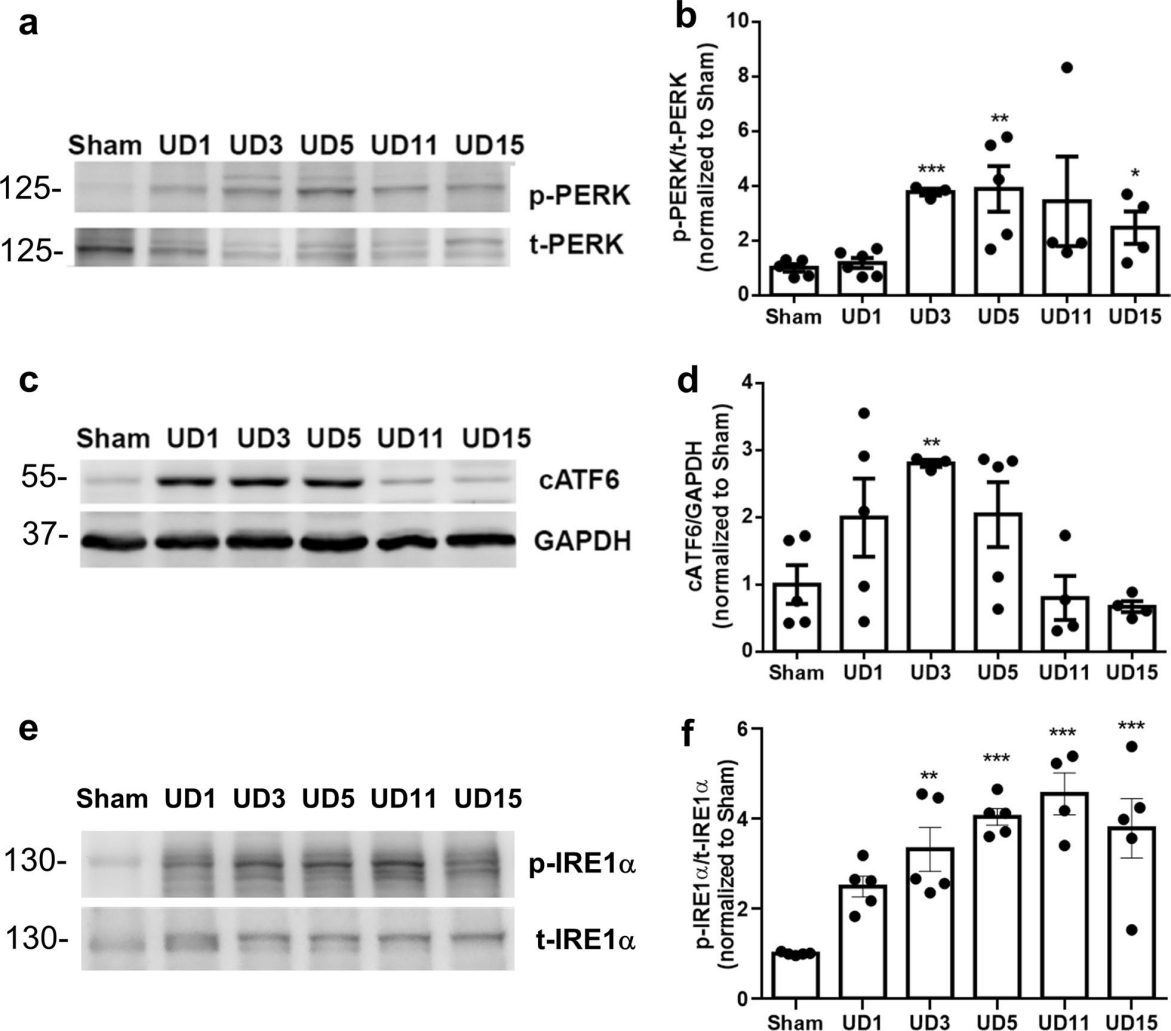
Activation of UPR signaling pathways after UIRI. **a** and **b** The expression of p-PERK and t-PERK in kidneys of UIRI mice were evaluated with western blot analysis and quantified. **c** UIRI induces the activation of ATF6 as demonstrated by enhanced expression of cleaved ATF6 (cATF6). GAPDH was used as an internal control. **d** Fold-change expression of cATF6 in kidneys of UIRI mouse as compared with that of sham group mouse. **e** and **f** The expression of p-IRE1 and t-IRE1 in mice kidneys were evaluated with western blotting and quantified. Data are expressed as means ± SEM, n = 3 ~ 6 in each group. *P < 0.05, **P < 0.01, and ***P < 0.001, as compared with sham group

Next, we monitored the activation status of UPR in the kidneys of the UIRI model. The results showed that the expressions of phospho-PERK and cleaved ATF6 (cATF6) were upregulated in UIRI kidneys at day 1 post-UIRI, and the peak timing of each molecule was around day 5 and day 3 post-UIRI, respectively (Fig. 1a–d). Similarly, the expression levels of phospho-IRE1α were upregulated in UIRI kidneys at day 1 post-UIRI (Fig. 1e). The expression of phospho-IRE1α in UIRI kidneys slightly decreased at day 15 post-UIRI, but remained substantially higher than sham controls (Fig. 1e). These findings suggest that the activation and modulation of UPR is an early response to UIRI and might influence the development of post-AKI fibrosis.

### The role of decreased XBP1u and XBP1s with post-AKI fibrosis

**Fig. 2:**
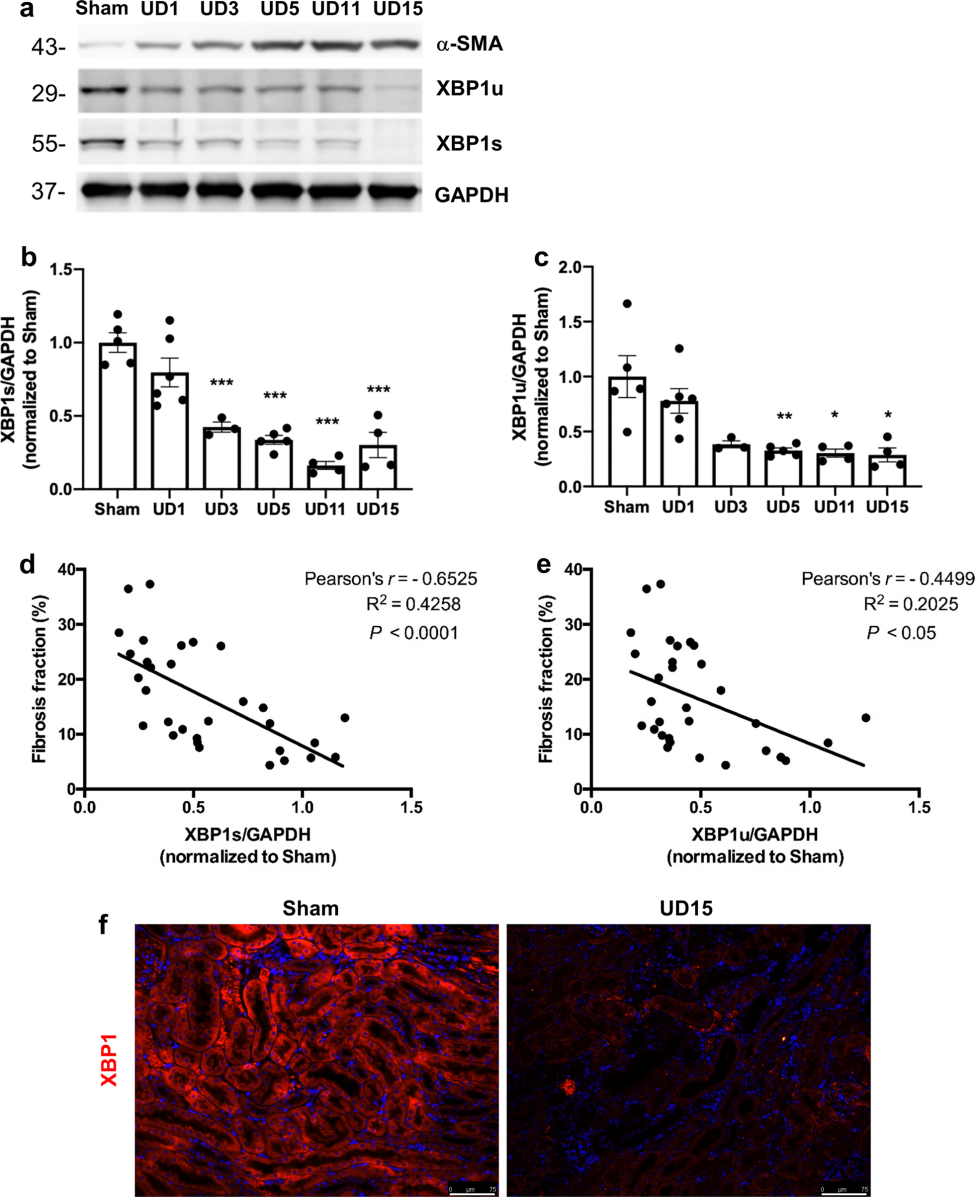
The expression of XBP1u and XBP1s was negatively correlated with post-AKI fibrosis. **a** Western blot analysis showed the expression of α-SMA, XBP1u, and XBP1s proteins. GAPDH was used as an internal control. **b** and **c** Quantification of fold-changes in expression levels of XBP1s (**b**) and XBP1u (**b**). Data are expressed as means ± SEM, n = 3 ~ 6 in each group. *P < 0.05, **P < 0.01, and ***P < 0.001, as compared with sham group. **d** and **e** Linear regression analysis of the correlation between fibrosis fraction and (**d**) XBP1s or (**e**) XBP1u protein expression in UIRI mice. **f** Representative images of XBP1 stained renal sections after 15 days of UIRI or Sham surgery. Scale bar: 75 μm

According to our previous finding, overwhelming of ER stress results in IRE1α phosphorylation and ER-associated degradation leads to selective XBP1u and XBP1s downregulation, which were associated with renal fibrosis progression in UUO model [17]. Hence, we further monitored the contribution of IRE1α to post-AKI fibrosis by examining the downstream splicing of XBP1. Both XBP1u and XBP1s protein expression levels were progressively declined following UIRI (Fig. 2a–c), especially in the dilated renal tubular section as shown in the immunofluorescence staining (Fig. 2f); XBP1s (Pearson’s r = − 0.6525, P < 0.001; Fig. 2d) and XBP1u protein expression (Pearson’s r = − 0.4499, P < 0.05; Fig. 2e) exhibited a significantly negative correlated with the degree of fibrosis. To comprehensively evaluate the expressions of different XBP1 isoforms in various kidney injury models, we measured α-SMA, XBP1u, and XBP1s expression in UUO and the adenine-induced CKD models. Upon model establishment, mice in UUO and adenine models had increased expression of α-SMA and decreased expression of XBP1s and XBP1u (Additional file 2: Fig. S2). Therefore, we propose that XBP1 downregulation can be a common pathway in post-AKI fibrosis.

### XBP1 knockout in renal proximal tubules leads to more severe kidney IRI

To specifically investigate the functional role of XBP1 in post-AKI fibrosis, we generated proximal tubular XBP1 conditional knockout (*XBP1*^*cKO*^, *Slc5a*^*CreERT2*^*; XBP1*^*fl/fl*^) mice using the cre/loxP system (Additional file 2: Fig. S3a). After 5 consecutive days of tamoxifen administration (75 mg/kg), the knockout efficacy was evaluated by IP injection of 500 ng/g ER stress inducer-Tunicamycin (TM, 500 ng/g) for 12 h to induce XBPs expression. And results were performed by western blot analysis and IF staining (Additional file 2: Fig. S3b, c). As Fig. S3b-c western blot analysis and immunofluorescence staining revealed that XBP1s expression was increased in XBP1^fl/fl^ treated with TM group and slightly decreased in XBP1^cKO^ treated vehicle group due to only proximal parts of tubular epithelial cells being knockout. While XBP1^cKO^ mice block TM-induced XBP1s expression. To specifically evaluated XBP1 deletion in the proximal tubular segment, we isolated the renal cortex and medulla fraction of XBP1 transgenic mice to detect XBP1 mRNA expression. The result showed that XBP1s mRNA expression decreased by approximately 65% in the cortex and 35% in the medulla as compared to XBP1^fl/fl^ cortex fraction (Additional file 2: Fig. S3d, e).

**Fig. 3:**
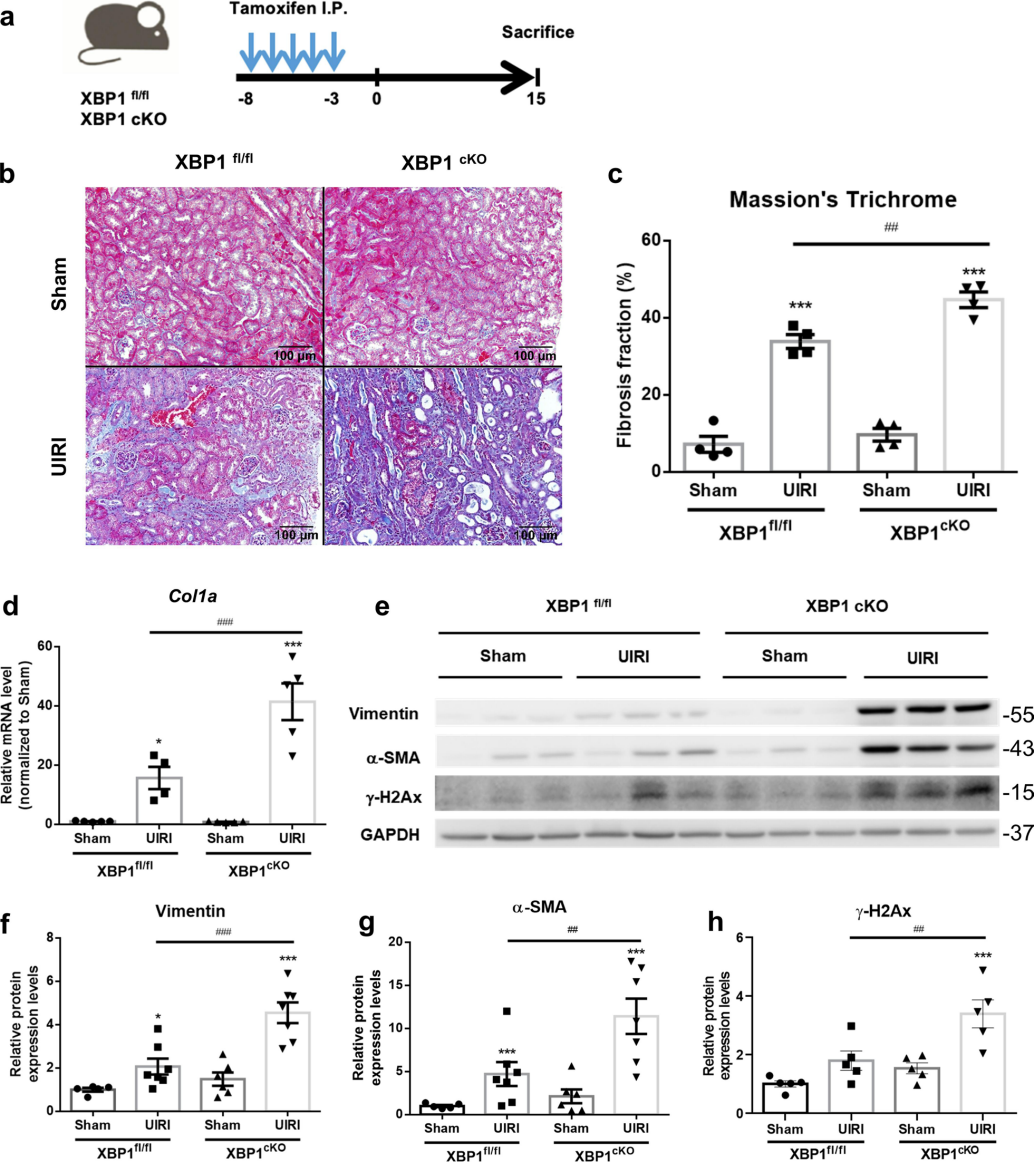
Specific proximal tubular knockout of XBP1 enhanced renal fibrosis in UIRI mice. **a** Diagram illustrates the timeline of the experiment. All the experimental mice were intraperitoneal injection of tamoxifen, then subjected to UIRI for 30 min. **b** Masson’s trichrome staining shows the increased fibrosis fraction in XBP1^cKO^ mice kidney section after UIRI. N = 3–4 for each group. Scale bar indicates 100 μm in 200×. **c** Quantitative scores of interstitial fibrosis were assessed. **d** qPCR assessment of the relative expression level of *Col1a* mRNA. **e**–**h** The expression of α-SMA and vimentin were examined with western blot analysis and quantified. N = 5–8 for each group. Data are expressed as means ± SEM. *P < 0.05, ***< 0.001 as compared with XBP1^fl/fl^ sham group, ^#^P < 0.05, ^##^P < 0.01, ^###^P < 0.001 as compared with the indicated group

To determine whether loss of XBP1 affected post injured renal fibrosis, XBP1^fl/fl^ and XBP1^cKO^ mice subjected to tamoxifen induction then underwent UIRI surgery and were sacrificed 15 days later (Fig. 3a). Masson’s trichrome staining of the injured kidney showed that the depletion of tubular XBP1 promoted post-UIRI fibrosis (Fig. 3b and c), and XBP1^cKO^ mice had more significant collagen deposition as evidenced by increased *Col1a* mRNA expression (Fig. 3d). Fibrotic markers, including α-SMA and vimentin, also significantly increased in XBP1^cKO^ mice subjected to UIRI compared to XBP1^fl/fl^ UD15 group(Fig. 3e–g). These data suggested that XBP1 deletion in proximal tubular exacerbated fibrosis development after IRI.

### XBP1 specific deletion in proximal tubular aggravates maladaptive repair process

**Fig. 4:**
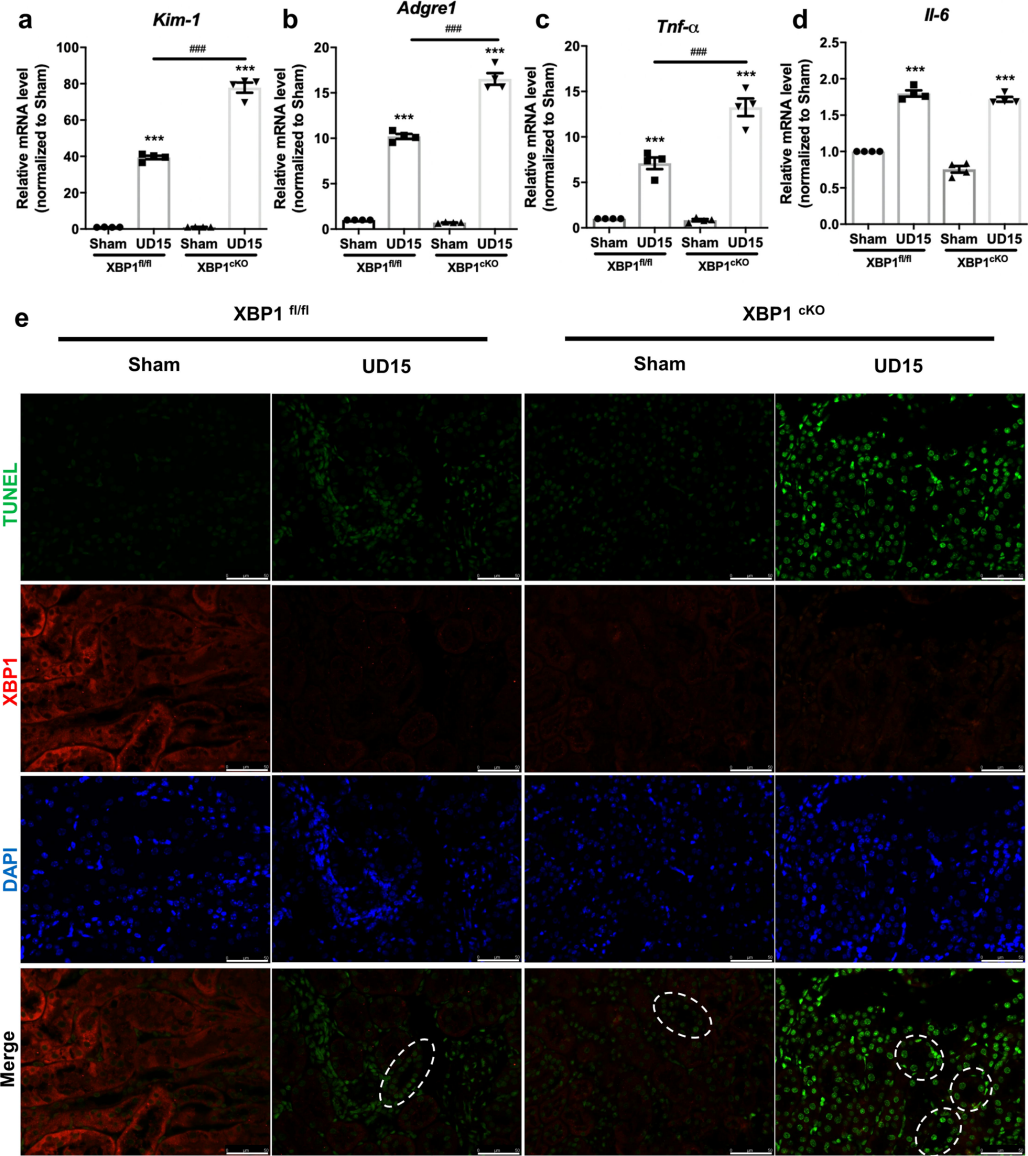
Proximal tubular-specific XBP1 knockout mice exacerbate maladaptive repaired mechanisms following UIRI. **a**–**d** qPCR assessment of *Kim-1, Adgre1, Tnf-α*, and *Il-6* mRNA expression levels. N = 6 for each group. Data are expressed as means ± SEM. ***P < 0.001 as compared with XBP1^fl/fl^ sham group, ^###^P < 0.001 as compared with the indicated group. **e** Representative images of TUNEL and XBP1 co-stained renal sections in XBP1^fl/fl^ or XBP1^cKO^ mice after 15 days of UIRI surgery. Selected areas indicated TUNEL positive tubules. Scale bar: 50 μm

Since progressive fibrosis is associated with the severity of the tubular injury, inflammatory response, and DNA damage response (DDR) [31, 32], the mRNA expression levels of *Kim-1* (kidney injury molecule-1), *Tnf-α*, and *Adgre1* (F4/80) were significantly increased in XBP1^cKO^ UD15 group compared to XBP1^fl/fl^ UD15 group but no difference in *Il-6* mRNA expression (Fig. 4a-d). To specifically investigate renal function impairment in UIRI model, we performed a contralateral nephrectomy in XBP1^cKO^ mice subjected to UIRI before one day of sacrifice (UNx, Additional file 2: Fig. S4a) due to the compensatory effect of the contralateral kidney, UIRI does not affect BUN and Scr levels, as previously described [30]. In comparison to XBP1^fl/fl^ UNx group, XBP1^cKO^ mice exposed to UNx demonstrated significantly higher BUN and Scr levels (Additional file 2: Fig. S4b and S4c). Furthermore, higher levels of the DNA damage marker γ-H2AX were found in XBP1^cKO^ mice with UIRI (Fig. 3h), and we performed TUNEL assay co-staining with XBP1 to examine the relationship between loss of XBP1 and DNA damage. As the results demonstrated that TUNEL positive areas were dissociated from XBP1 stained sections, and the injured tubular in XBP1^cKO^ UD15 group had more DNA fragmentation stained than the XBP1^fl/fl^ UD15 group (Fig. 4e). Collectively, these findings confirm that XBP1^cKO^ mice were more vulnerable to IRI and impaired the subsequent repair processes.

### Loss of XBP1 leads to cell cycle G2/M arrest

Recent studies place emphasis on the contribution of injured proximal tubule cells arrest in the G2/M phase to the progression from AKI to CKD, possibly through producing profibrogenic growth factors [10]. We identified similarly the phenomenon of cell cycle G2/M arrest in our UIRI model, evidenced by an elevated protein expression of Chk1, p21, and Ki67^+^pHH3^+^ tubular epithelial cells after UIRI (Additional file 2: Fig. S5a-e). Moreover, Ki67^+^pHH3^+^ areas were obviously increased in XBP1^cKO^ compared to XBP1^fl/fl^ both in sham and UIRI groups (Additional file 2: Fig. S5f-g), suggesting XBP1 deletion in tubular cells prolongs cell cycle arrest in G2M phase and contributes to fibrosis progression.

**Fig. 5:**
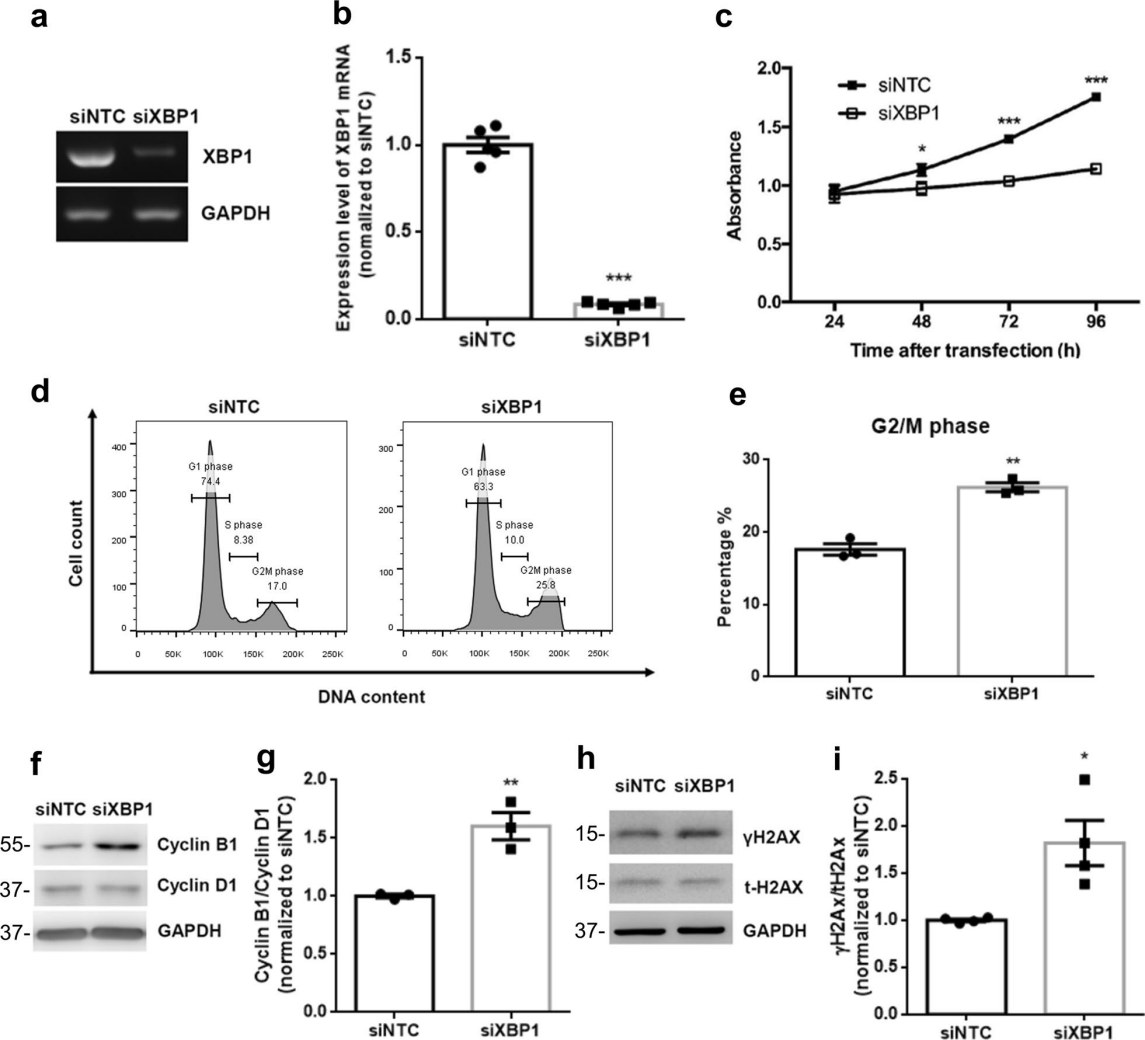
Knockdown of XBP1 induces cell cycle G2/M arrest in HK2 renal tubule epithelial cells. **a** and **b** The knockdown efficiency of XBP1 was determined by reverse transcription-PCR and quantified. siNTC: negative control siRNA. **c** Loss of XBP1 results in growth inhibition. MTS assay was conducted every 24 h for up to 4 days to examine the effect of XBP1 deficiency on the growth rate of cells based on the absorbance. **d** Loss of XBP1 results in modulating the cell cycle. Cells were transfected with siNTC or siXBP1. After 24 h, cells were harvested and subjected to flow cytometric analysis to determine the cell cycle distribution. **e** The amounts of cells in the G2/M phase of the cell cycle in HK2 cells following transfection of indicated siRNA were quantified. **f** Western blot analysis showed the expression of cyclin B1 and cyclin D1 proteins. GAPDH was used as an internal control. **g** The ratio of cyclin B1 to cyclin D1 in HK2 cells following transfection of indicated siRNA. **h** Western blot analysis showed the expression of γH2AX and t-H2AX proteins. **i** The ratio of γH2AX to t-H2AX in HK2 cells following transfection of indicated siRNA. Data are expressed as means ± SEM of three independent experiments. *P < 0.05, **P < 0.01, and ***P < 0.001, as compared with siNTC group

Next, we further investigated whether XBP1 down-regulation was functionally connected with cell cycle regulation. Human renal tubular epithelial cells HK2 were transfected with either scrambled siRNA (siNTC) or siRNA duplexes against XBP1 (siXBP1), and the silence efficiency was validated by semi-quantitative PCR (Fig. 5a and b). An apparent cell growth inhibition in XBP1-deficient cells compared to scrambled controls was demonstrated by the MTS assay (Fig. 5c). We then assessed the cell cycle status in XBP1-deficient cells using flow cytometry and found that the percentage of cells in G2/M phase increased in XBP1-silenced cells compared to that in scrambled siRNA-transfected cells (Fig. 5d and e). XBP1-silenced cells exhibited higher cyclin B1/cyclin D1 ratios than scramble controls (Fig. 5f and g). As G2M phase arrest is typical of DDR, we then investigated the potential role of XBP1 in DDR. γH2AX, a marker of DNA damage, significantly increased in XBP1 knockdown cells (Fig. 5h and i)*.* Collectively, these results provide evidence that XBP1 is essential for proper cell cycle progression.

### The profibrotic paracrine effect of XBP1 silenced HK2 cells

**Fig. 6:**
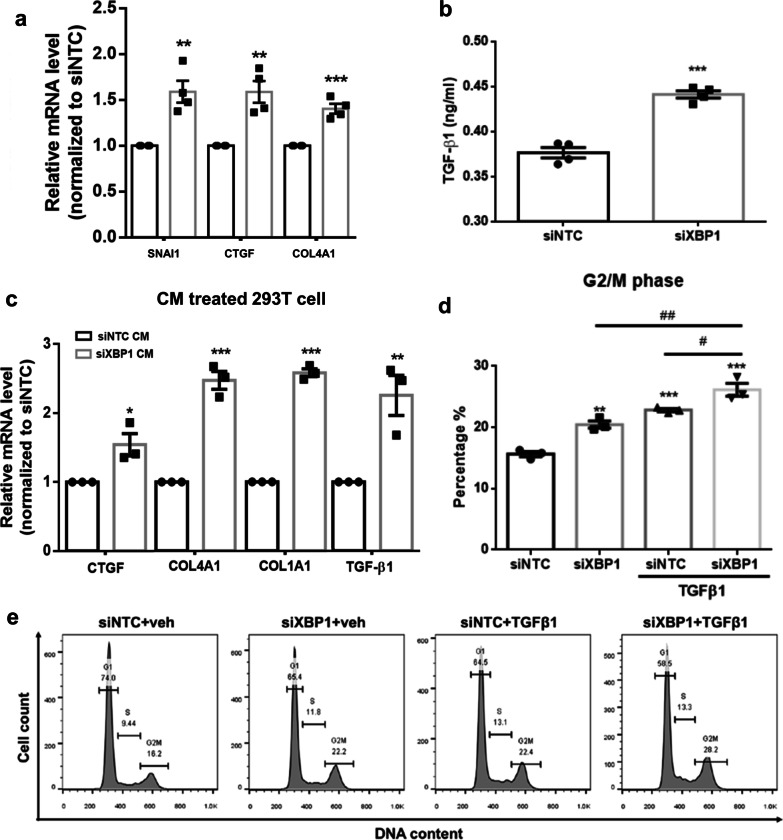
XBP1-deficient HK2 cells possess profibrotic properties. **a** XBP1 deficiency enhances the mRNA expression of *SNAI1, CTGF* and *COL4A1* in HK2 cells. **b** Compared to cells transfected with siNTC, XBP1-deficient HK2 cells secrete elevated levels of active TGF-β1 as assessed by ELISA. **c** Conditioned medium from XBP1-deficient HK2 induces mRNA expression of *CTGF, COL4A1, COL1A1*, and *TGFβ1* in 293 T cells. **d** Loss of XBP1sensitizes HK2 cells to TGFβ1-induced cell cycle G2/M arrest. HK2 cells were transfected with indicated siRNA, and 48 h later cells were treated with vehicle (veh) or TGFβ1 (5 ng/mL) for 24 h. Cells were then harvested and subjected to flow cytometric analysis to determine the cell cycle distribution. **e** The amounts of cells in the G2/M phase of the cell cycle were quantified. Data are expressed as means ± SEM of three independent experiments. *P < 0.05, **P < 0.01, and ***P < 0.001, as compared with siNTC group. ^##^P < 0.01 compared between indicated groups

Maladaptive repair mediated partial-EMT contributes to G2M arrested tubular cells, increasing profibrotic cytokine production, supporting the role of tubular epithelial cells in renal fibrosis initiation and progression [9–11]. Therefore, we examined related genes expression and found *SNAI1*, *CTGF*, and *COL4A1* mRNA expression significantly upregulated in XBP1-deficient cells compared to scramble controls (Fig. 6a). In addition, the amount of secreted active TGFβ1 determined by ELISA, the master regulator of fibrosis, also increased in the culture media from XBP1-deficient cells (Fig. 6b). 293 T human embryonic kidney cells with related undifferentiated phenotypes were used to evaluate the stimulating effect of TGFβ1 released from XBP1 silenced HK-2 conditioned medium (CM). After exposure to siXBP1 CM, 293 T cells exhibited higher expression of fibrotic-associated genes, including TGFβ1, CTGF, COL4A1, and COL1A1, than those of siNTC CM treated cells (Fig. 6c). These results simulate a paracrine effect of XBP1-deficient tubular epitheliums on adjacent renal cells. Furthermore, cumulating studies support the profibrotic effects of TGFβ1 in human kidney diseases. Thus, we investigated the effect of XBP1 deficiency on TGFβ1-mediated profibrotic signaling. HK2 cells were transfected with either siNTC or siXBP1 for 48 h, followed by treatment with vehicle or 5 ng/mL of TGFβ1 for 24 h. These findings reveal that TGFβ1-treated XBP1-deficient cells display an increased G2/M population compared with TGFβ1-treated controls (Fig. 6d, e). Taken together, the presented data show that XBP1 deletion enhanced TGFβ1 secretion in tubular cells, resulting in a vicious cycle that stimulates surrounding cells prolonged cell cycle arrest and profibrotic factors production.

### Trap1, a cell cycle-regulated protein, is identified as downregulated in XBP1 deficiency model

**Fig. 7:**
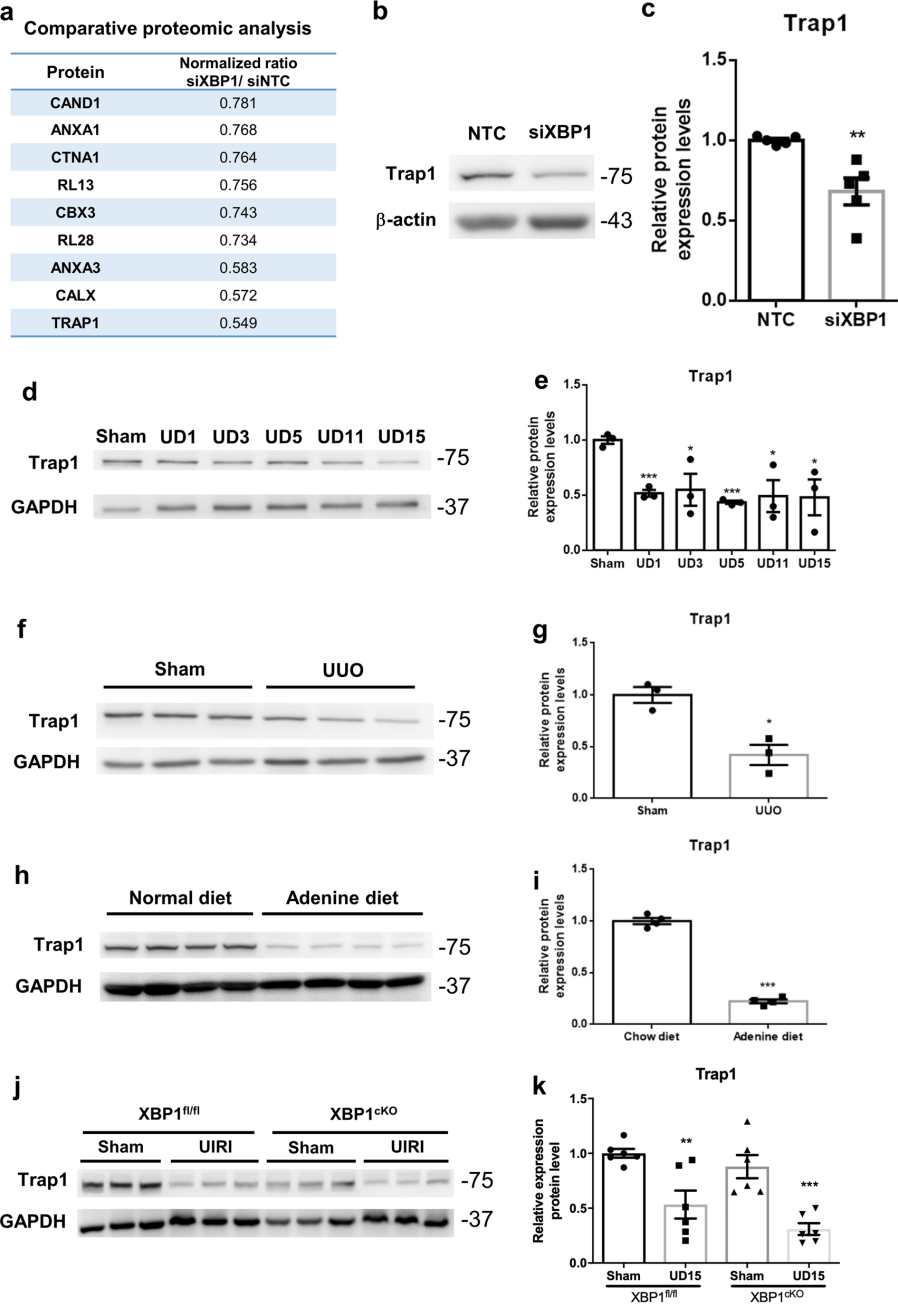
Cell cycle-regulated protein Trap1 is downregulated in the loss of XBP1 models. **a** Protein expression levels in XBP1 deficient HK-2 were presented by MS-based proteomics analysis. The present data shows siXBP1 over siNTC protein expression normalized ratio. **b** and **c** The protein expression of Trap1 in XBP1 silenced HK-2 cell was examined by western blot and quantified. GAPDH was used as an internal control. Data are expressed as means ± SEM of three independent experiments. *P < 0.05. **d** and **e** The protein expression of Trap1 in mice kidneys of UIRI was evaluated with western blotting and quantified. **f** and **g** The protein expression of Trap1 in mice kidneys of UUO was evaluated with western blotting and quantified. **h** and **i** The protein expression of Trap1 in mice kidneys of adenine diet was evaluated with western blotting and quantified. **j** and **k** The protein expression of Trap1 in XBP1^cKO^ mice kidneys was evaluated with western blotting and quantified. Data are expressed as means ± SEM of at least three independent measurements. *P < 0.05, and ***P < 0.001, as compared with sham group

We found XBP1 is essential for HK-2 cells as silencing XBP1 causes cell maladaptive repaired phenotype. Furthermore, XBP-1 is a critical signaling molecule that participated in adaptive UPR; loss of XBP-1 would disrupt downstream effector proteins, making it difficult to maintain the biological processes. Accordingly, we performed proteomic analysis to examine differentially expressed proteins in HK-2 cells silenced of XBP1 compared to scramble control. Among 378 candidate proteins, 26 were up-regulated and 13 were down-regulated (Fig. 7a and Additional file 3). To determine the functional regulator affected by the loss of XBP1, we searched for all of the significantly changed proteins involved in cell cycle control or fibrosis progression. TNF receptor-associated protein1 (Trap1), a member of the heat shock protein 90 family that participates in cell-cycle regulation [33, 34] and protects against renal tubulointerstitial fibrosis in UUO model [35], was found to be the most reduced protein in our proteomic analysis data. We further validated Trap1 protein expression correspondingly decreased in HK-2 cells silenced with XBP-1 (Fig. 7b and c). Besides, Trap1 protein expression was also evaluated in the low XBP1 expression mice kidney samples, which have been validated in Fig. 2 and Additional file 2: Fig. S2. Findings from UIRI, UUO, and adenine models showed that Trap1 protein decreased in all experimental groups, paralleling the loss of XBP-1 protein (Fig. 7d–i). In addition, XBP1^cKO^ mice also showed relatively lower Trap1 expression both in sham group and UIRI group compared to XBP1^fl/fl^ (Fig. 7j and k). These results support a highly regulatory possibility between XBP1 and Trap1.

### Spliced XBP1 transcriptionally regulates Trap1 expression

**Fig. 8:**
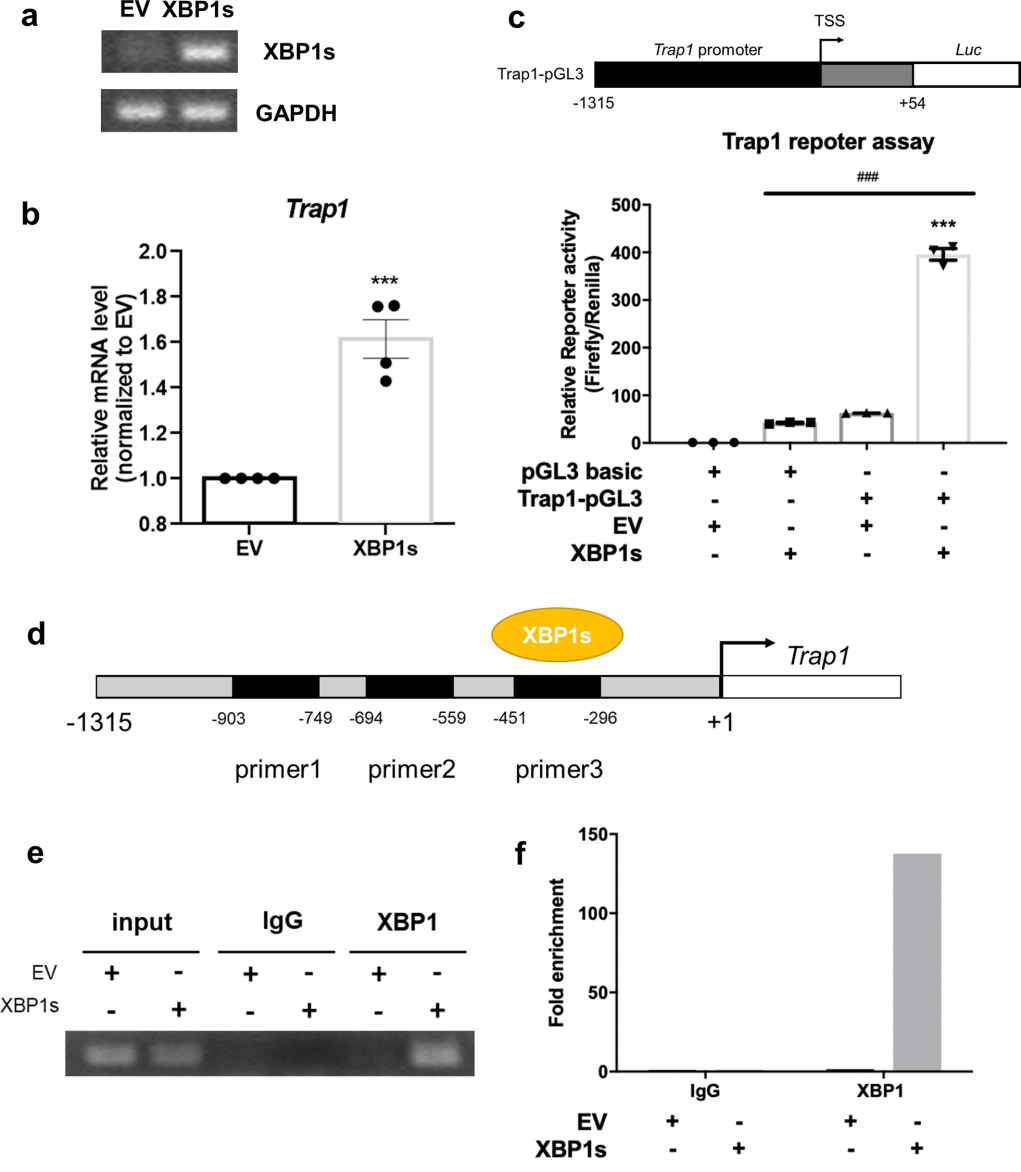
Spliced XBP1 participated in Trap1 expression at transcriptional regulation. **a** XBP1s mRNA expression levels were determined by semi-quantitative PCR. HK-2 cells were transfected with 1ug of pLAS3W-XBP1s. **b** Trap1 mRNA expression was evaluated by qPCR in HK-2 cells overexpressed with XBP1s. N = 4 for each group. Data are expressed as means ± SEM. ***P < 0.0001 as compared with empty vector (EV). **c** Trap1 promoter binding ability of XBP1 was examined by luciferase reporter assay. Upper panel: schematic diagrams of Trap1 promoter-luciferase reporter. Lower panel: relative luciferase activity in HK-2 cells transfected with a Trap1 promoter-luciferase reporter plasmid or empty vector (pGL3 basic), along with EV or XBP1s plasmid. Renilla luciferase activity was used as an internal control and for normalization of transfection efficiency. N = 3 for each group. Data are expressed as means ± SEM. **d**–**f** ChIP-PCR assay was performed to analyze the binding of XBP1s to Trap1 promoter in 293 T cells. **d** Three sets of primer pairs covered the − 296 ~ − 451, − 559 ~ 694, − 749 ~ 903 regions, and qPCR showed that XBP1s bound to − 296 ~ − 451 region (**e** and **f**)

According to our findings (Figs. 2 and 7), Trap1 expression correlated with those of XBP1. We subsequently explored whether Trap1 was regulated by XBP1 through manipulating XBP1s expression. Trap1 mRNA was upregulated in HK-2 cells transfected with the XBP1s plasmid (Fig. 8a, b). To more specifically investigate XBP1s promoter binding activity, Trap1 promoter sequence was cloned into pGL3-basic plasmid (Fig. 8c). We showed that HK-2 cells transfected with pGL3-Trap1 promoter, pRenilla luciferase, and pAs3w-XBP1s demonstrated a substantial increase in luciferase activity. Moreover, the binding site of XBP1s on Trap1 promoter was confirmed by the ChIP-qPCR assay. Only the Trap1 promoter primer set (Region 3, − 296 ~ − 451) can amplify the signaling from XBP1s antibody pull-down chromatin when HK-2 cells were transfected with XBP1s plasmid (Fig. 8d–f). These results suggest that XBP1s transcriptionally regulates Trap1 expression.

### Trap1 overexpression rescues silenced XBP1 induced G2M arrest

**Fig. 9:**
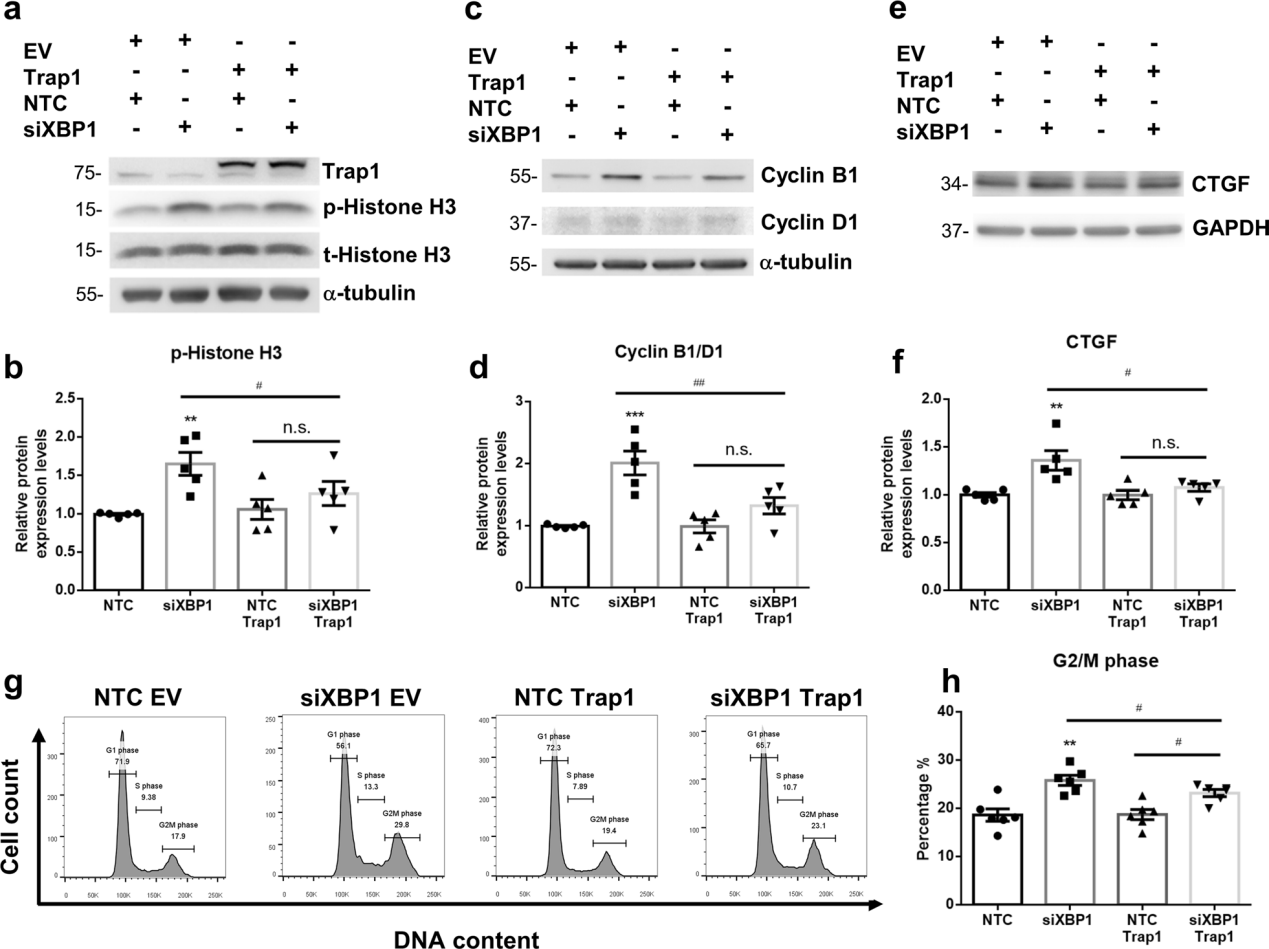
Trap1 overexpression rescues XBP1 knockdown induced cell cycle G2M arrest and profibrotic factor expression. **a** and **b** Trap1 and phospho-Histone H3 protein expression were examined with western blot and quantified in HK2 cells following transfection of indicated siRNA and plasmid. Total-Histone H3 and a-tubulin were used as an internal control. **c** and **d** Cyclin B1 and cyclin D1 protein expression ratios were examined with western blot and quantified. **e** and **f** CTGF protein expression ratio was examined with western blot and quantified. GAPDH as internal control. N = 4–6 for each group. Data are expressed as means ± SEM. **P < 0.01 as compared with siNTC group. ^#^P < 0.05 as compared with indicated two groups. **g** and **h** Overexpression of Trap1 rescues knockdown XBP1 induced cell cycle arrest. Cells were transfected with indicated siRNA and plasmid. After 48 h, cells were harvested and subjected to flow cytometric analysis to determine the cell cycle distribution. N = 4 for each group. Data are expressed as means ± SEM. **P < 0.01 as compared with siNTC group

As Trap1 has been reported to regulate cell cycle and protect mitochondrial integrity [33], we are curious about whether restoring Trap1 expression would relieve cell cycle arrest. Trap1 overexpression did not affect cell cycle G2M arrest markers, histone H3 phosphorylation and cyclinB1/D1 ratio expressions. However, Trap1 overexpression in XBP-1 silenced group significantly decreased histone H3 phosphorylation and cyclinB1/D1 protein expression compared to siXBP-1 group (Fig. 9a–d). Consistent with western blot results, Trap1 overexpression attenuated silenced XBP1-induced cell cycle arrest in G2M phase (Fig. 9g and h). Furthermore, profibrotic factor production was also relieved in Trap1 restoration group, evidenced by CTGF protein expression levels in XBP1 deficient and Trap1 overexpression group compared to the XBP1 silenced group (Fig. 9e and f). These results indicate that Trap1 is a regulator capable of preventing the prolongation of cell cycle G2M arrest and reducing profibrotic factor production in XBP-1 deficient HK-2 cells.

## Discussion

AKI is an important contributor to CKD and ESRD [36]. Patients with AKI are at an increased risk of progressive CKD during hospitalization between 2011 and 2012, according to the United States Renal Data System (USRDS) [4]. Since proximal tubular epithelia exhibit high oxygen demand and contain low antioxidants [37], they are often thought to be the main targets of AKI. During the transition from AKI to CKD, injured tubular epithelial cells play an important role. Partial-EMT, cell-cycle arrest, and imbalanced proteostasis are important factors in post-injury fibrosis [38]. EMT damages renal parenchyma by retarding tubular cell cycle progression, resulting in renal function decline [11]. The production of profibrogenic factors by injured tubular epithelial cells stimulate fibroblast proliferation and collagen synthesis. Furthermore, the association between partial-EMT mediated tubular epithelial cells cell-cycle arrest and renal fibrosis has also been reported [9]. The pathophysiologic role of proteostatic imbalance in injured kidney is supported by in vivo findings of kidney fibrosis amelioration by chemical chaperones or ER stress preconditioning [16, 18, 39]. While these factors exaggerate fibrosis, it is unknown how they interact.

**Fig. 10:**
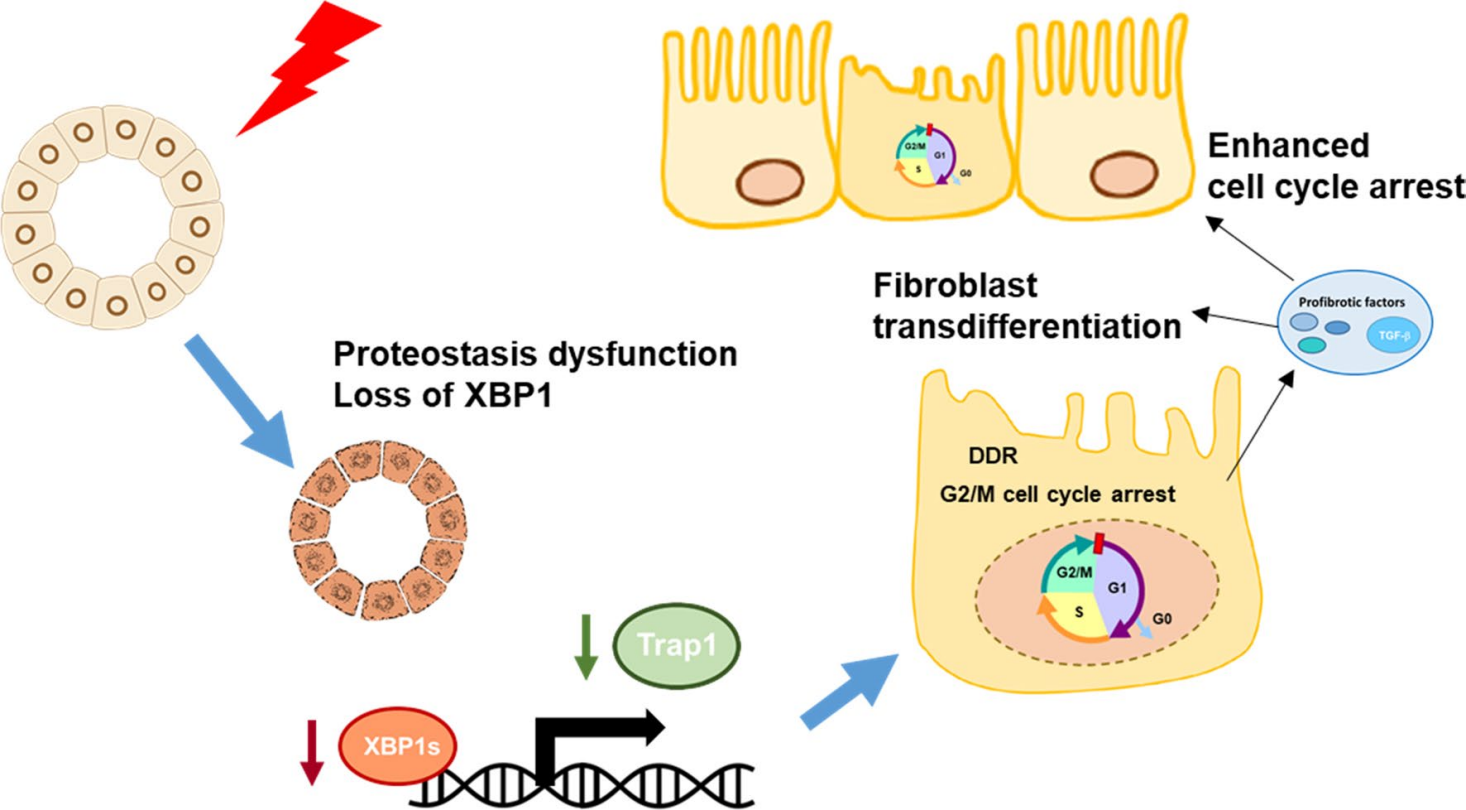
The mechanism for the involvement of the XBP1-Trap1 axis in post-AKI fibrosis. Our results show that AKI induces UPR activation as demonstrated by increased expression of p-PERK, cATF6, and p-IRE1. However, the expression of XBP1 is downregulated and is coincident with decreased expression of Trap1 and elevated expression of α-SMA and fibrosis progression. XBP1 directly regulates Trap1 expression. Restoration of Trap1 expression can alleviate the loss of XBP1 causes prolonged G2/M cell cycle arrest, relieves profibrotic factors expression, and ameliorates renal fibrosis progression

The present study showed that AKI promoted post-injury renal fibrosis in a mouse UIRI model, based on findings of increased expressions of EMT marker, α-SMA, and fibrosis severity on Masson trichrome staining. These results are in accordance with previous findings using both unilateral and bilateral IRI models [40, 41]. In addition, we found that UPR initiators PERK, IRE1α, and ATF6 were activated following AKI, while the expression of XBP1 gradually lowered throughout experiments and inversely correlated with the degree of renal fibrosis. We further demonstrated that losing XBP1 caused G2/M cell cycle arrest and inhibited proliferation of proximal tubule epithelial cells. Our findings uncover the role of ER stress master regulator XBP1, which connect cell cycle regulation to the development of renal fibrosis (Fig. 10).

Modulating the expression of XBP1s or XBP1u and its protective role has been reported previously. An ATF6-dependent maladaptive ER response induced by impaired XBP1s nuclear translocation was observed in diabetic nephropathy [42]. In comparison with the control group, nuclear expressions of XBP1s are lower in both murine models of diabetic nephropathy and diabetic patients. Furthermore, the IRE1α-XBP1 pathway is activated in co-chaperone Sec63-deficient tubule epithelial cells. Double knockout of Sec63 and Xbp1 enhances the severity of polycystic kidney disease in mice and re-expression of XBP1s is able to rescue the inactivation of Sec63 with XBP1 or IRE1α induced interstitial inflammation and fibrosis [43, 44]. Fan and Xiao et al. showed that the expressions of XBP1s decreased in the kidneys of patients with AKI. Although there was no statistical difference in the amount of XBP1s between stage III AKI patients with and without progression to CKD, a trend toward lower XBP1s expressions in patients with progressive AKI could be observed [15]. Collectively, these findings support the notion that loss of XBP1 potentially contributes to the progression of renal diseases.

As the previous study identified that the hypomorphic variants of XBP1 are the susceptibility factors to spontaneous inflammatory bowel disease [45]; and progressive CKD development is associated with intensive renal inflammation cytokines: *Il-6, Tnf-α*,* Adgre1,* and kidney injury molecule *Kim-1* expression. Therefore, we detected the mRNA expression in renal tissue and found that only *Il-6* mRNA expression levels did not significantly upregulate in XBP1^cKO^ UIRI group compared to XBP1^fl/fl^ UIRI group, which could be explained that XBP1s can activate IL-6 mRNA transcription in cancer cell [46, 47].

Conversely, sustained activation of XBP1s could lead to unfavored responses. Overexpression of XBP1s, for example, has been shown to enhance apoptosis and atherosclerosis progression in human umbilical vein endothelial cells (HUVECs) [48]. Moreover, Moe et al*.* demonstrated that XBP1s is implicated in Lipopolysaccharide (LPS) induced AKI and inflammation; and manipulating XBP1s overexpress in tubular epithelium can potentiate prolonged LPS exposure induced inflammatory cytokines and kidney injury molecules expression. Although previous studies have shown XBP1s protect against inflammatory disorders like IBD and polycystic kidney disease, Moe’s work highlights the unique role of XBP1s in tubular epitheliums during LPS-induced sepsis.

Given the inconsistent effect of genetic manipulation of XBP1 in disease therapy, the current study applied a high throughput screening approach to identify non-toxic molecules that selectively activate the IRE1α-XBP1s arm of the UPR without globally activating other signaling pathways related to ER stress [49]. IXA4, the most selective IRE1α-XBP1 activator, can promote the degradation of Alzheimer’s disease-associated amyloid precursor protein (APP) mutants, prevent APP-associated mitochondrial dysfunction, and remodel obesity-induced metabolic dysfunction [50]. Based on these encouraging publications and our present findings, the therapeutic effect of IXA4 on AKI to CKD transition is under our investigation and selective activation of IRE1α-XBP1s signaling in IRI would be clarified in further research.

IRE1α signaling activates XBP1s expression, while our finding showed that IRE1α phosphorylation without corresponding XBP1 upregulation but inhibition in the UIRI model. Our previous research showed that overwhelming ER stress induction in UUO with IRE1α-phosphorylation and ER-associated degradation results in the suppression of XBP1u and XBP1s protein expression, implying the adaptive UPR is disrupted [17]. Similarly, Dufey et al*.* demonstrated that genotoxic stress arouses IRE1α activation independent of canonical XBP1 splicing, leading to regulated IRE1α-dependent decay of mRNA (RIDD) mediated DDR and cell cycle arrest [51]. miRNA also participated in XBP1 regulation. Duan et al*.* showed that XBP1 is the direct target for miR-214 in the animal model of cardiac hypertrophy and heart failure [52, 53]. Additionally, another study presented by Denby et al*.* indicated that miR-214 expression is increased in the UUO model [54], in which XBP1 expression is downregulated in our study [17]. These findings indicate the possible mechanism involved in IRE1α phosphorylation without XBP1 splicing. While, XBP1 deficiency or decreased nuclear expressions of XBP1 have been shown to contribute to pancreatic β cells dysfunction and keratinocytes senescence through activating IRE1α [55, 56]. Depleting XBP1 induces the hyper-activation of IRE1α, leading to an impaired insulin secretion of β-cells [55]. Such a negative-feedback mechanism also participates in oncogenic H-Ras-induced premature senescence and subsequent tumor development [56]. Mechanisms through which the activated IRE1α is involved in the pathogenesis of renal diseases remain unknown. Several plausible mechanisms have been suggested, including the cleavage of ER-localized mRNAs and microRNAs through regulating RIDD and through activating downstream effectors such as JNK. The link between JNK activation and renal injuries has been supported by findings that JNK co-opted with other signaling pathways to promote cell death, inflammation, and fibrosis [57].

Currently, the protective effect of XBP1 downregulation against I/R-induced kidney injury has been reported by Zhang et al*.* [58]. In the study, the renal function and pathological score significantly attenuated in XBP1 heterozygous mice subjected to I/R-induced AKI and a higher survival rate compared to the wild-type I/R group. Different from their approach, we used proximal tubular specific XBP1 conditional knockout mice to highlight the pathogenetic roles of XBP1 downregulation in renal epithelial, aggravating post-injured renal fibrosis. Furthermore, the profibrotic phenotypes of tubular cells were also observed in HK-2 with silenced XBP1, such as cell cycle arrest and TGF β1 secretion. Hence, our data support the concept that loss of XBP1 aggravates renal fibrosis.

XBP1s act as an adaptive UPR transcriptional regulator which participates in biological functions including helper T cell activation, myoblasts differentiation, and chondrocyte differentiation. Although researchers have identified potential targets of XBP1, the functional role of most of these XBP1 target genes remain poorly characterized. Our works demonstrated that Trap1 was one of the downstream targets of XBP1. Trap1 is one of the chaperons belonging to heat shock protein 90 family, which is located in mitochondrial and participates in maintaining mitochondrial function, proteostasis, and cell cycle progression [33]. Recent studies revealed that TRAP1 silencing results in cell cycle G2M phase progression in cancer cells [59–61]. In addition, overexpression of Trap1 can attenuate UUO-induced renal fibrosis and maintain mitochondrial integrity [35]; however, the underlying mechanism is still unclear. In our study, we revealed that the loss of XBP1 and Trap1 expression is consistent across different kidney injury models. On top of that, Trap1 is one of XBP1 downstream targets, which is able to relieve XBP1 silencing induced cell cycle arrest and decrease profibrotic factors production.

It has been considered that cell cycle arrest regulated by DDR is a protective mechanism to ensure that injured cells restore their functions [32]. However, prolonged cell cycle arrest caused by severe AKI may lead to maladaptive repair. Yang et al. showed that the transition of cell cycle S arrest to G2/M arrest had been observed in tubular cells of acute aristolochic acid nephropathy and various ischemic AKI models, reflecting a switch from an active repair response to a maladaptive repair [10]. Clearly, the increased probability of G2/M arrest may precipitate profibrotic factor production in damaged tubular epithelial cells. In our study, we demonstrated that the anti-fibrotic property of Trap1 was associated with its ability to limit genome instability. Growing evidence links XBP1 to the regulation of cell cycle and DNA repair [62–65]. For instance, deletion of XBP1 in yeast impeded DNA repair process [64], inhibited pseudohyphal growth on nitrogen-limited agar media due to the high expression of mitotic cyclin gene CBL2 [62]. Furthermore, XBP1 also has a role in IRE1α-mediated upregulation of cyclin A, which in turn enhances cell proliferation in various prostate cancer cell lines [63]. Recently, Wang and Chao et al. showed that XBP1 silencing increased apoptosis and reduced cell proliferation in mouse granulosa cells. They found that the increase in cyclin E1 and the decrease in cyclin A1 and cyclin B1 contributed to S phase cell cycle arrest in XBP1-deficient cells [65]. These findings suggest that XBP1 plays a central role in cell fate determination when cells face various stimuli in different cells.

## Conclusions

The present study identified that the downregulation of XBP1 was profibrotic, and the process was mediated through autocrine and paracrine regulations in combination. The XBP1-Trap1 axis is an instrumental mechanism responsible for post-AKI fibrosis, which is a novel regulatory pathway.

## Supplementary Information

**Additional file 1: Table S1.** Primer sequences of PCR, real-time PCR, and genotyping.

**Additional file 2: Figure S1.** UIRI causes prominent renal damage and development of fibrosis. (a) Diagram illustrates the timeline of the experiment. The left kidney of male C57BL/6 mice was subjected to renal ischemia/reperfusion injury (UIRI) and then sacrificed at different days as indicated. UDx: x days after UIRI. (b) PAS staining represents the accumulation of debris in the tubular lumen after UIRI. The arrowhead in the lower panel indicates debris. Scale bar indicates 200 μm in 40x, 50 μm in 200x. (c) qPCR assessment of the relative expression level of *Kim-1* mRNA. (d) Masson’s trichrome staining shows the increased fibrosis fraction in kidney section after UIRI. Scale bar indicates 50 μm in 200x. (e) Quantitative scores of interstitial fibrosis were assessed. (f and g) The expression of α-SMA was examined with western blot analysis and quantified. Data are expressed as means ± SEM, n = 3 ~ 6 in each group. * P < 0.05 and *** P < 0.001, as compared with sham group. **Figure S2.** Loss of XBP1 expression is a universal characteristic in renal fibrosis models. (a) Western blot analysis showed the protein expression of α-SMA, XBP1u and XBP1s in UUO mice model. GAPDH was used as an internal control. (b-d) Quantification of relative protein expression levels of α-SMA, XBP1u and XBP1s. (e) Western blot analysis showed the protein expression of α-SMA, XBP1u and XBP1s in adenine diet mice model. GAPDH was used as an internal control. (f–h) Quantification of relative protein expression levels of α-SMA, XBP1u and XBP1s. N = 3–4 for each group, * P < 0.05, ** P < 0.01, and *** P < 0.001, as compared with sham or chow diet group. **Figure S3.** Proximal tubular conditional knockout mice blocked XBP1s activation. (a) Diagram illustrates SLC5a^CreERT2^; XBP1^fl/fl^ mice. (b) After tamoxifen administration, mice were subjected to IP injection of 500 ng/g of Tunicamycin for 12 h. Western blot analysis showed protein expression of XBP1s after Tunicamycin induction in XBP1^fl/fl^ or XBP1^cKO^ mice. GAPDH was used as an internal control. (c) Immunofluorescence staining demonstrated XBP1 expression in Tunicamycin treated mice kidneys. Scale bar: 250 μm. (d) XBP1s mRNA expression level was determined by semi-quantitative PCR. (e) qPCR assessment of the relative expression level of XBP1s mRNA. **Figure S4. **Proximal tubular XBP1 specific knockout mice were vulnerable to UIRI-induced kidney injury. (a) Diagram illustrates the experimental timeline of tamoxifen administration and UIRI with contralateral nephrectomy (Nx) surgery in XBP1^fl/fl^ and XBP1^cKO^ mice. (b and c) Blood urea nitrogen (BUN) and serum creatinine (Scr) levels were measured after 1 day of contralateral Nx. N = 3 for each group. ** P < 0.01, and *** P < 0.001, as compared with XBP1^fl/fl^ Sham group. **Figure S5. **UIRI induces cell cycle arrest in G2/M phase. (a-d) The expression of chk1 and p21 in mice kidneys was evaluated with western blotting and quantified. GAPDH was used as an internal control. Data are expressed as means ± SEM, n = 3 ~ 6 in each group. * P < 0.05, ** P < 0.01, and *** P < 0.001, as compared with sham group. (e and f) Representative images of Ki67^+^ pHH3^+^ renal sections in (e) WT mice or (f) XBP1^cKO^ and XBP1^fl/fl^ mice subjected to UIRI or Sham operation. Selected areas indicated highly expressed double-positive tubules. Scale bar: 50 μm. (g) Number of Ki67^+^pHH3^+^ tubular cells. Data are expressed as means ± SEM. * P < 0.05, ** P < 0.01, and *** P < 0.001, as compared with XBP1^fl/fl^ sham group. ^###^ P < 0.001 compared between indicated groups.

**Additional file 3.** Proteomic analysis of differentially expressed proteins in HK-2 cells silenced of XBP1 compared to scramble control.

## Data Availability

The datasets used and/or analyzed during the current study are available from the corresponding author on reasonable request.

## Abbreviations

AKI: Acute kidney injury
APP: Amyloid precursor protein
ATF6: Activating transcription factor 6
Bip: Binding immunoglobulin protein
BUN: Blood urea nitrogen
CHOP: CAAT/enhancer-binding protein (C/EBP) homologous protein
CKD: Chronic kidney disease
CM: Conditioned medium
CTGF: Connective tissue growth factor
cKO: Conditional knockout
DDR: DNA damage response
eIF2α: Eukaryotic translation initiation factor 2α
EMT: Epithelial-mesenchymal transition
ESRD: End-stage renal stage
ER: Endoplasmic reticulum
ERAD: ER associated protein degradation
EV: Empty vector
IBD: Inflammatory bowel disease
IRE1α: Inositol-requiring enzyme 1α
JNK: C-Jun N-terminal kinase
LPS: Lipopolysaccharide
NTC: Negative control
Nx: Nephrectomy
PERK: PKR-like ER kinase
RIDD: Regulated Ire1 dependent decay
Scr: Serum creatinine
TGFβ1: Transforming growth factor β 1
Trap1: TNF receptor associate protein 1
UIRI: Unilateral ischemia–reperfusion injury
UNx: UIRI with contralateral Nx
UPR: Unfolded protein response
UUO: Unilateral ureteral obstruction
XBP1: X binding protein-1
XBP1s: Spliced isoform of XBP1
XBP1u: Unspliced isoform of XBP1
