# Supplementary material for: The down-regulation of XBP1, an unfolded protein response effector, promotes acute kidney injury to chronic kidney disease transition
Source: J Biomed Sci. 2022 Jun 28;29:46. doi: 10.1186/s12929-022-00828-9 (PMC9241279; doi:10.1186/s12929-022-00828-9)
Supplement: Supplementary file 1 — Additional file 1: Table S1. Primer sequences of PCR, real-time PCR, and genotyping. [file 12929_2022_828_MOESM1_ESM.docx]

**The down-regulation of XBP1, an unfolded protein response effector, promotes acute kidney injury to chronic kidney disease transition**

**Chen et al. Additional file 1**

**Supplementary Table S1. Primer sequences of PCR, real-time PCR, genotyping, and plasmid construction.**

| **Semi quantitative PCR primer** | | | |
| --- | --- | --- | --- |
| Name | Species |  | Sequence |
| XBP-1 | Human | Forward | gTCTgCTgAgTCCgCA |
|  |  | Reverse | TCCTTCTgggTAgACCTCTgggAg |
| GAPDH | Human | Forward | TTCACCACCATggAgAAggC |
|  |  | Reverse | ggCATggACTgTggTCATgAg |
| **Real-time PCR primer** | | | |
| CTGF | Human | Forward | gTggAgTATgTACCgACggC |
|  |  | Reverse | gCAggCACAggTCTTgATgA |
| COL4A1 | Human | Forward | gAAgggTTTCCCCggATTC |
|  |  | Reverse | CCCgACTgTCCCgTTATgC |
| COL1A1 | Human | Forward | CgCATgggTCTTCAAgCAAg |
|  |  | Reverse | TgACCAACCgAACATgACCA |
| GAPDH | Human | Forward | TgCACCACCAACTgCTTAgC |
|  |  | Reverse | ggCATggACTgTggTCATgAg |
| Trap1 | Human | Forward | AgCgCACTCATCAggAAACT |
|  |  | Reverse | CgCATgAACAggCCgTAATC |
| TGFβ-1 | Human | Forward | TgACgTCACTggAgTTgTACgg |
|  |  | Reverse | ggTTCATgTCATggATggTgC |
| Promoter of Trap1 (Region 1) | Human | Forward | gATgATgggTTTTCACCATgT |
|  |  | Reverse | CTTTCTgATTggCAATTggTT |
| Promoter of Trap1 (Region 2) | Human | Forward | TCCACACggAACCAACCTAT |
|  |  | Reverse | AgggAggCACAgTCTCAAAA |
| Promoter of Trap1 (Region 3) | Human | Forward | ggTggTATTggTCACTgggT |
|  |  | Reverse | gTgTCTgCgACCTTTgTgAA |
| XBP1s | Human/Mouse | Forward | ggTCTgCTgAgTCCgCAgCAgg |
|  |  | Reverse | gAAAgggAggCTggTAAggAAC |
| Kim-1 | Mouse | Forward | ACATATCgTggAATCACAACgAC |
|  |  | Reverse | ACAAgCAgAAgATgggCATTg |
| Col1a | Mouse | Forward | CTCCTCTTAggggCCACT |
|  |  | Reverse | CCACgTCTCACCATTgggg |
| Adgre1 | Mouse | Forward | gCCACggggCTATgggATgC |
|  |  | Reverse | ACCCACAgTgTCCAggCAAgg |
| Il-6 | Mouse | Forward | ACCgCTATgAAgTTCCTCTC |
|  |  | Reverse | CCTCTgTgAAgTCTCCTCTC |
| Tnf-α | Mouse | Forward | CCACCACgCTCTTCTgTCTAC |
|  |  | Reverse | AgggTCTgggCCATAgAACT |
| GAPDH | Mouse | Forward | TgACTCCACTCACggCAAAT |
|  |  | Reverse | TCTCgCTCCTggAAgATgg |

| **Genotyping** | | |
| --- | --- | --- |
| Slc5a^CreERT2^ | Forward | CTAAACATgCTTCATCgTCggTC |
|  | Reverse | TCTgACCAgAgTCATCCTTAgCg |
| XBP1 ^fl/fl^ | Forward | CACCCTAAggCCCAgTTTgC |
|  | Reverse_WT | ACACgAgAgAgTCCCAATCC |
|  | Reverse_KO | CCTCgAAgCTTATCgATACC |
| **Plasmid construction** | | |
| NheI-Human XBP1s | Forward | ATCggCTAgCATggATTATAAggATgACgACgATAAAgTggTggTggCAgCCgCg |
| NheI-Human XBP1s | Reverse | CgATgCTAgCTTAgACACTAATCAgCTggggAAAgAgTTC |
| KpnI-Human Trap1 | Forward | ATTAggTACCATggCgCgCgAgCTgCg |
| EcoR1-Human Trap1 | Reverse | ATgAgAATTCCAgTgTCgCTCCAgggCC |
| NheI-Human Trap1 promoter | Forward | ATTAgCTAgCCATCCggTACAggTATgT |
| HindIII-Human Trap1 promoter | Reverse | ATCgAAgCTTggACCgTACACgATgggAA |
