# Supplementary material for: The down-regulation of XBP1, an unfolded protein response effector, promotes acute kidney injury to chronic kidney disease transition
Source: J Biomed Sci. 2022 Jun 28;29:46. doi: 10.1186/s12929-022-00828-9 (PMC9241279; doi:10.1186/s12929-022-00828-9)
Supplement: Supplementary file 2 — Additional file 2: Figure S1. UIRI causes prominent renal damage and development of fibrosis. (a) Diagram illustrates the timeline of the experiment. The left kidney of male C57BL/6 mice was subjected to renal ischemia/reperfusion injury (UIRI) and then sacrificed at different days as indicated. UDx: x days after UIRI. (b) PAS staining represents the accumulation of debris in the tubular lumen after UIRI. The arrowhead in the lower panel indicates debris. Scale bar indicates 200 μm in 40x, 50 μm in 200x. (c) qPCR assessment of the relative expression level of Kim-1 mRNA. (d) Masson’s trichrome staining shows the increased fibrosis fraction in kidney section after UIRI. Scale bar indicates 50 μm in 200x. (e) Quantitative scores of interstitial fibrosis were assessed. (f and g) The expression of α-SMA was examined with western blot analysis and quantified. Data are expressed as means ± SEM, n = 3 ~ 6 in each group. * P < 0.05 and *** P < 0.001, as compared with sham group. Figure S2. Loss of XBP1 expression is a universal characteristic in renal fibrosis models. (a) Western blot analysis showed the protein expression of α-SMA, XBP1u and XBP1s in UUO mice model. GAPDH was used as an internal control. (b-d) Quantification of relative protein expression levels of α-SMA, XBP1u and XBP1s. (e) Western blot analysis showed the protein expression of α-SMA, XBP1u and XBP1s in adenine diet mice model. GAPDH was used as an internal control. (f–h) Quantification of relative protein expression levels of α-SMA, XBP1u and XBP1s. N = 3–4 for each group, * P < 0.05, ** P < 0.01, and *** P < 0.001, as compared with sham or chow diet group. Figure S3. Proximal tubular conditional knockout mice blocked XBP1s activation. (a) Diagram illustrates SLC5aCreERT2; XBP1fl/fl mice. (b) After tamoxifen administration, mice were subjected to IP injection of 500 ng/g of Tunicamycin for 12 h. Western blot analysis showed protein expression of XBP1s after Tunicamycin induction in XBP1fl/fl or XBP1cKO [file 12929_2022_828_MOESM2_ESM.docx]

**The down-regulation of XBP1, an unfolded protein response effector, promotes the transition from acute kidney injury to chronic kidney disease**

**Chen et al. Additional file 2**

**Supplementary Figures**

**
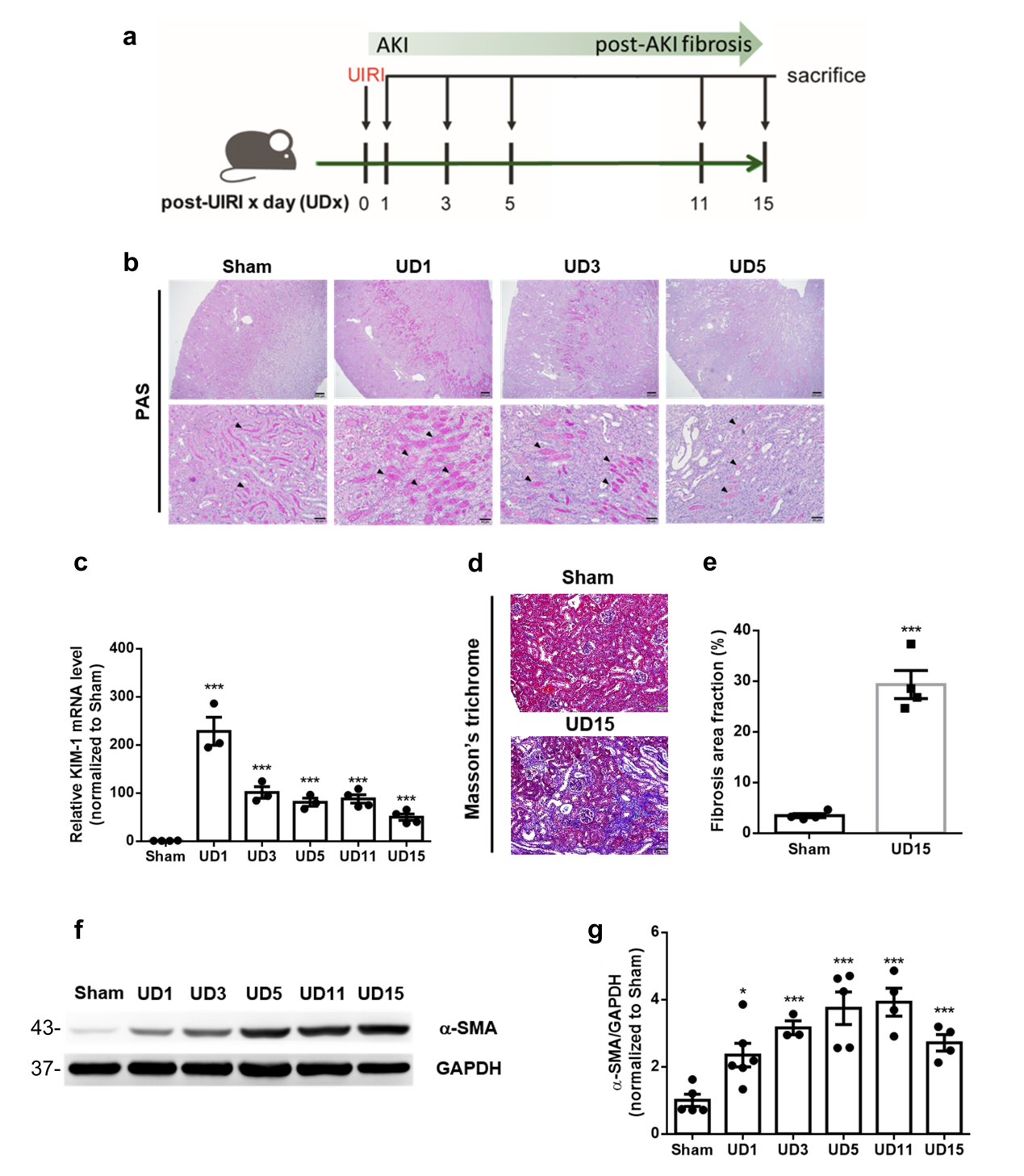
Figure S1. UIRI causes prominent renal damage and development of fibrosis.**

(a) Diagram illustrates the timeline of the experiment. The left kidney of male C57BL/6 mice was subjected to renal ischemia/reperfusion injury (UIRI) and then sacrificed at different days as indicated. UDx: x days after UIRI. (b) PAS staining represents the accumulation of debris in the tubular lumen after UIRI. The arrowhead in the lower panel indicates brush border and debris, respectively. Scale bar indicates 200 μm in 40x, 50 μm in 200x. (c) qPCR assessment of the relative expression level of *Kim-1* mRNA. (d) Masson’s trichrome staining shows the increased fibrosis fraction in kidney section after UIRI. Scale bar indicates 50 μm in 200x. (e) Quantitative scores of interstitial fibrosis were assessed. (f and g) The expression of a-SMA was examined with western blot analysis and quantified. Data are expressed as means ± SEM, n=3~6 in each group. * P < 0.05 and *** P < 0.001, as compared with sham group.

**
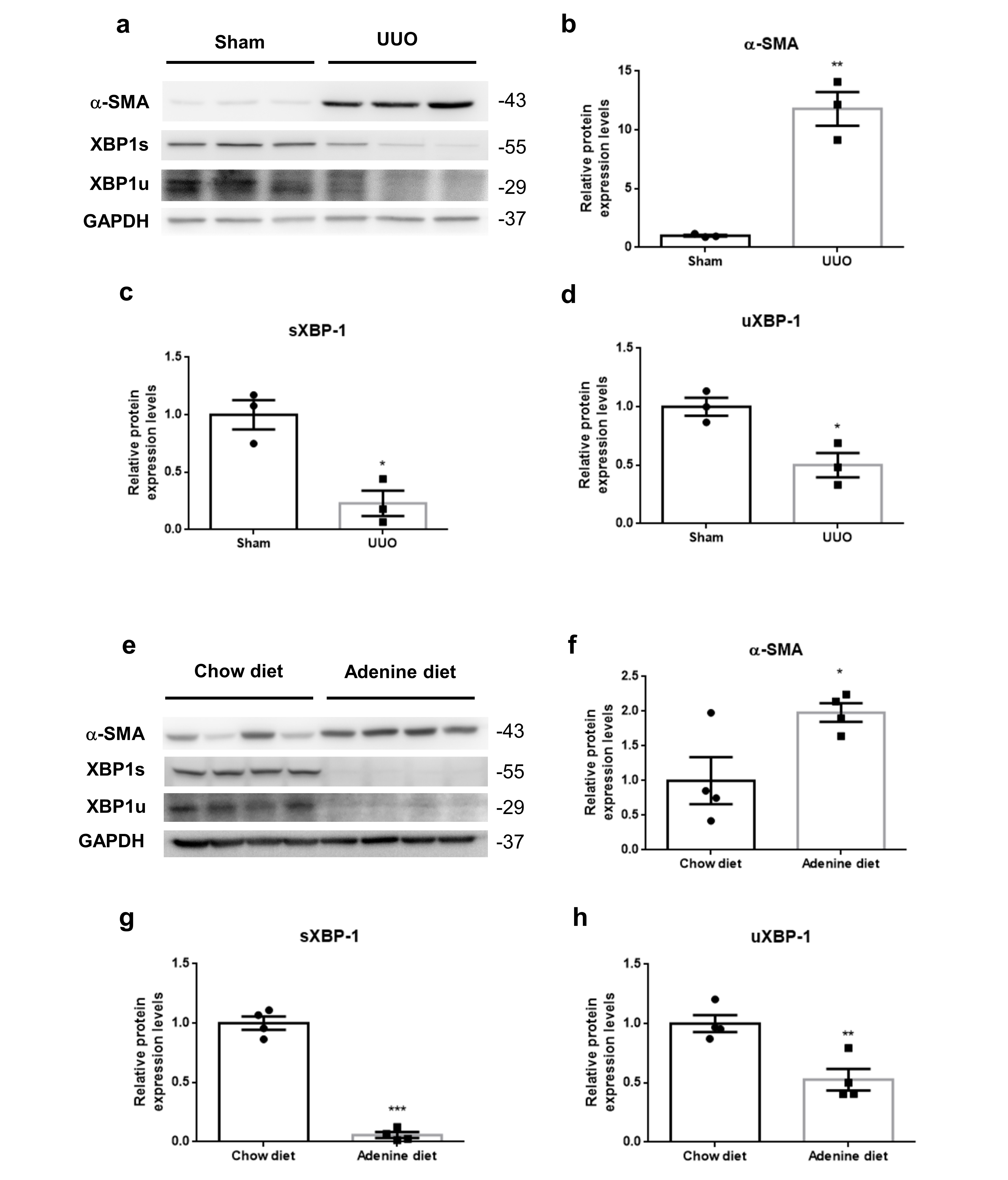
**

**Figure S2. Loss of XBP1 expression is a universal characteristic in renal fibrosis models.**

(a) Western blot analysis showed the protein expression of α-SMA, XBP1u and XBP1s in UUO mice model. GAPDH was used as an internal control. (b-d) Quantification of relative protein expression levels of α-SMA, XBP1u and XBP1s. (e) Western blot analysis showed the protein expression of α-SMA, XBP1u and XBP1s proteins in adenine diet mice model. GAPDH was used as an internal control. (f-h) Quantification of relative protein expression levels of α-SMA, XBP1u and XBP1s. N=3-4 for each group, * P < 0.05, ** P < 0.01, and *** P < 0.001, as compared with sham or chow diet group.


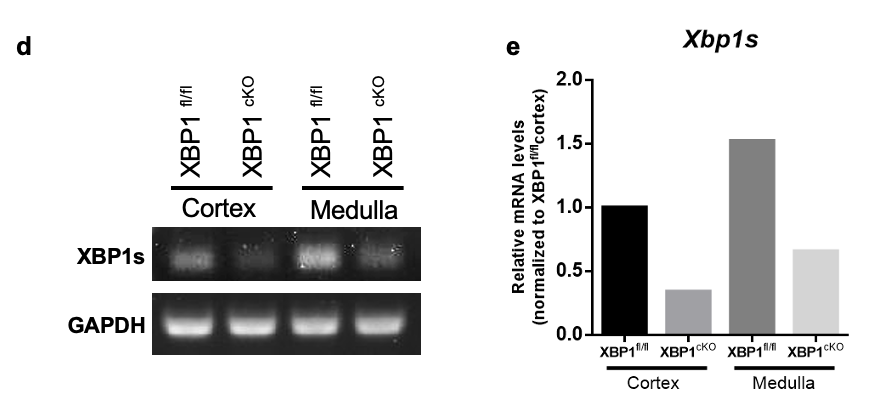
**
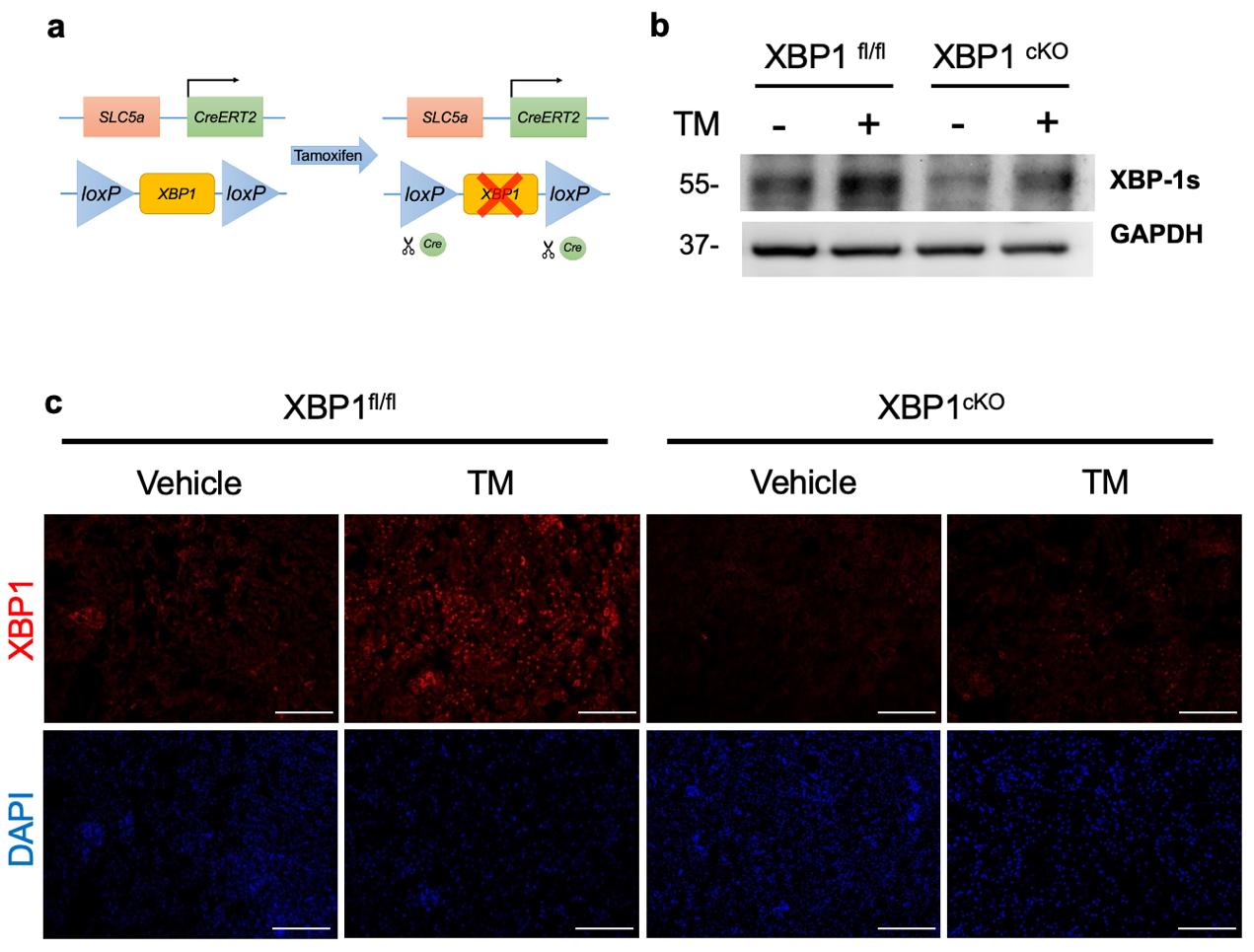
Figure S3. Proximal tubular conditional knockout mice blocked XBP1s activation.**

(a) Diagram illustrates SLC5a^CreERT2^; XBP1^fl/fl^ mice. (b) After tamoxifen administration, mice were subjected to IP injection of 500 ng/g of Tunicamycin for 12 hours. Western blot analysis showed protein expression of XBP1s after Tunicamycin induction in XBP1^fl/fl^ or XBP1^cKO^ mice. GAPDH was used as an internal control. (c) Immunofluorescence staining demonstrated XBP1 expression in Tunicamycin treated mice kidneys. Scale bar: 250 μm. (d) XBP1s mRNA expression level was determined by semi-quantitative PCR. (e) qPCR assessment of the relative expression level of XBP1s mRNA.

**
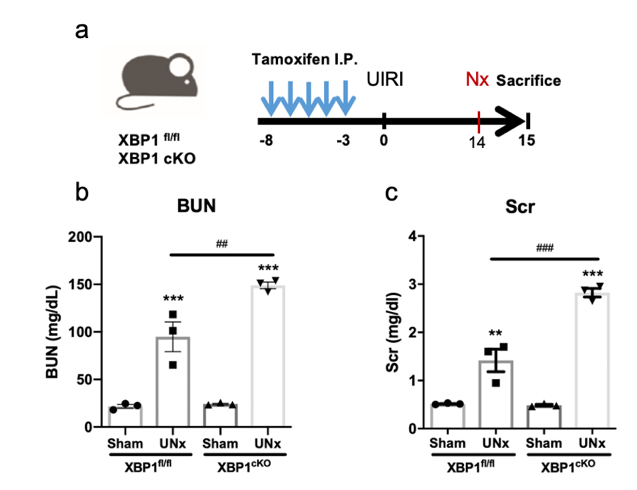
Figure S4. Proximal tubular XBP1 specific knockout mice were vulnerable to UIRI-induced kidney injury.**

(a) Diagram illustrates the experimental timeline of tamoxifen administration and UIRI with contralateral nephrectomy (Nx) surgery in XBP1^fl/fl^ and XBP1^cKO^ mice. (b and c) Blood urea nitrogen (BUN) and serum creatinine (Scr) levels were measured after 1 day of contralateral Nx. N=3 for each group. ** P < 0.01, and *** P < 0.001, as compared with XBP1^fl/fl^ Sham group.

**
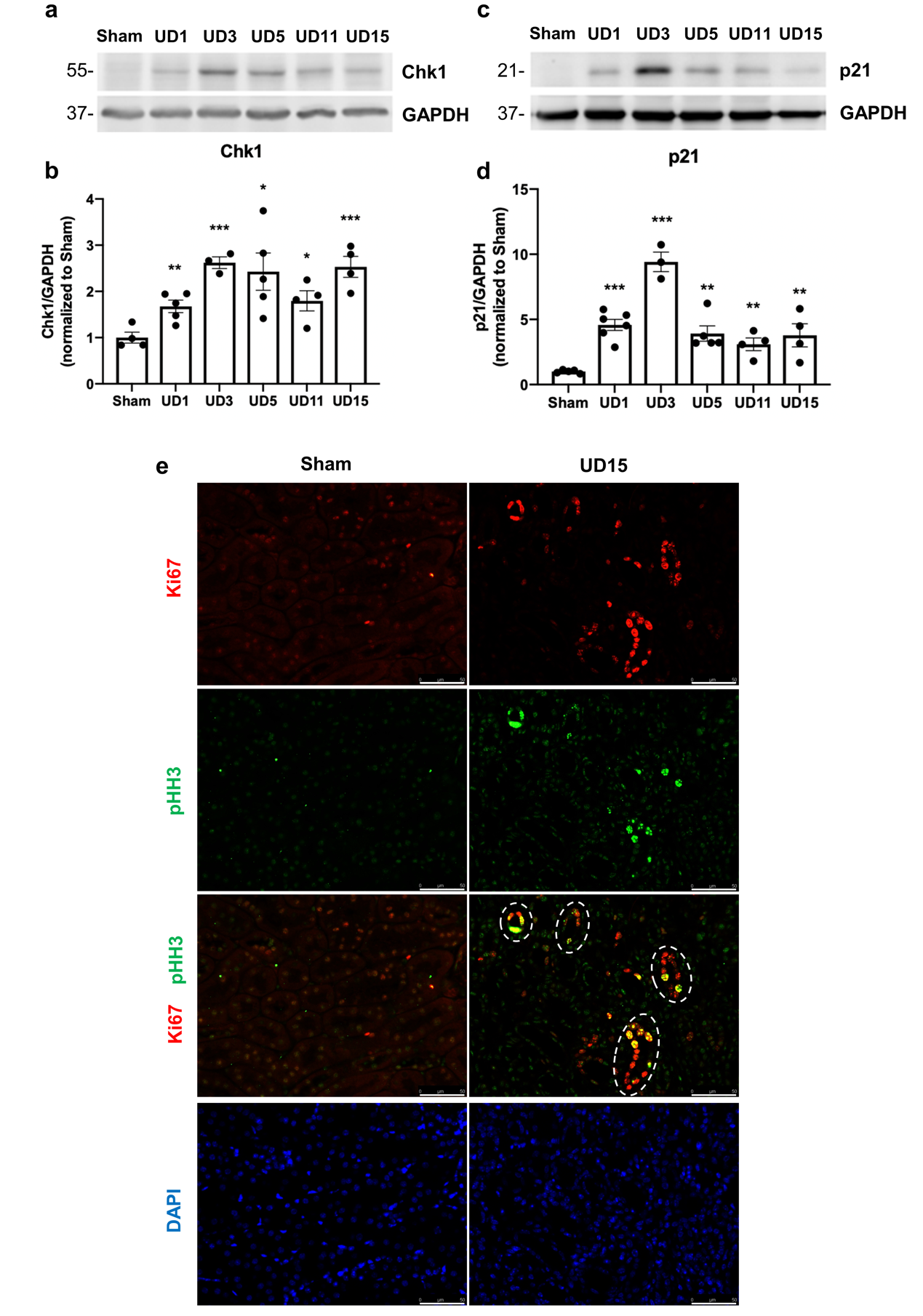
**

**

Figure S5. UIRI induces cell cycle arrest in G2/M phase.**

(a-d) The expression of chk1 and p21 in mice kidneys was evaluated with western blotting and quantified. GAPDH was used as an internal control. Data are expressed as means ± SEM, n=3~6 in each group. * P < 0.05, ** P < 0.01, and *** P < 0.001, as compared with sham group. (e and f) Representative images of Ki67^+^ pHH3^+^ renal sections in (e) WT mice or (f) XBP1^cKO^ and XBP1^fl/fl^ mice subjected to UIRI or Sham operation. Selected areas indicated highly expressed double-positive tubules. Scale bar: 50 μm.
